# Multiscale cytometry and regulation of 3D cell cultures on a chip

**DOI:** 10.1038/s41467-017-00475-x

**Published:** 2017-09-07

**Authors:** Sébastien Sart, Raphaël F.-X. Tomasi, Gabriel Amselem, Charles N. Baroud

**Affiliations:** Laboratory of Hydrodynamics (LadHyX)-Department of Mechanics, Ecole Polytechnique, CNRS-UMR7646, 91128 Palaiseau, France

## Abstract

Three-dimensional cell culture is emerging as a more relevant alternative to the traditional two-dimensional format. Yet the ability to perform cytometry at the single cell level on intact three-dimensional spheroids or together with temporal regulation of the cell microenvironment remains limited. Here we describe a microfluidic platform to perform high-density three-dimensional culture, controlled stimulation, and observation in a single chip. The method extends the capabilities of droplet microfluidics for performing long-term culture of adherent cells. Using arrays of 500 spheroids per chip, in situ immunocytochemistry and image analysis provide multiscale cytometry that we demonstrate at the population scale, on 10^4^ single spheroids, and over 10^5^ single cells, correlating functionality with cellular location within the spheroids. Also, an individual spheroid can be extracted for further analysis or culturing. This will enable a shift towards quantitative studies on three-dimensional cultures, under dynamic conditions, with implications for stem cells, organs-on-chips, or cancer research.

## Abstract (web summary)

3D cell culture is more relevant than the two-dimensional format, but methods for parallel analysis and temporal regulation of the microenvironment are limited. Here the authors develop a droplet microfluidics system to perform long-term culture of 3D spheroids, enabling multiscale cytometry of individual cells within the spheroid.

## Introduction

Preserving functional cellular phenotype is essential for many biotechnology applications such as drug screening, disease modeling or tissue engineering. This has led to growing interest in developing technologies adapted for three-dimensional (3D) cultures, and spheroids in particular^1–5^, since 3D culture regulates numerous important functions that are significantly altered in monolayers (2D)^6, 7^. However, inherent difficulties in maintaining and manipulating the spheroids have hindered access to high-throughput, quantitative measurements of the cell behavior. Instead, typical protocols for obtaining such data rely on using flow cytometry on the dissociated cells, which loses all information on the relationship between a phenotype and the cell location within the 3D culture. In parallel, powerful microscopy and image analysis methods have been developed for understanding the structural organization within the spheroids, but they are limited in throughput^8, 9^.

The current approaches for producing spheroids include traditional batch methods, including spinner flasks or low-attachment plates^10^. These protocols yield a large number of spheroids but with limited control on the size distribution and the culture environment^11^. More recent developments have used micro-fabrication to provide a bottom-up approach in which cells are aggregated together in controlled conditions (e.g., AggreWell™ plates, InSphero GravityPLUS Technology)^12–14^. However, while these systems allow medium exchange for modulating the culture conditions, the procedure is labor intensive and cannot be parallelized without the use of complex robotic systems.

These limitations have motivated the implementation of 3D culture methods within microfluidic channels as a way to remedy the shortcomings of the existing approaches^15^. Indeed, the use of microfluidics leverages the tools that have been developed for flow control and observation on chips, such as the ability to generate a spatially or temporally variable concentration of biomolecules^16^. This has led to several microfluidic proofs of concept for producing the spheroids, either in flowing droplets^1, 17–19^ or within microfabricated wells on a chip^20, 21^. The long-term spheroid culture and observation have recently been demonstrated using wells in the microchannel floor, which allow for perfusion controlled multi-condition stimulation and in situ analysis^2^. However, these platforms have only been demonstrated for modest numbers of spheroids and the analysis remains limited to measuring mean behaviors. In contrast, droplet methods are particularly attractive since they provide a scalable way of encapsulating and confining samples^22, 23^, while offering a wide range of manipulation tools^22, 24, 25^.

In this general context there is a strong need for a high functionality platform for controlled 3D cell cultures. Indeed, the next generation platforms would ideally integrate a wide range of capabilities in a single device, including (1) the production of the spheroids, (2) their maintenance in a viable and productive state, (3) the control and modulation of their environment (e.g., bring a stimulus/drug), (4) the staining and observation of single cells in situ, and (5) the selective recovery of any spheroid of interest for further analysis or culture. Such a platform would not only increase the throughput of high-content screening methods, it would also enable qualitatively new experiments by providing access to completely new protocols.

In this paper, we show how droplet microfluidics can be extended to provide high-density 3D cultures on a chip, by leveraging several technologies for drop manipulation^22^ and combining them with the gelation of the droplets to allow long-term culture and single-cell observations. The platform yields quantitative characterization on the population scale, but also on the scale of thousands of individual spheroids and hundreds of thousands of cells in situ within their spheroid, while allowing the extraction of a single spheroid for further analysis. This simultaneous increase in throughput and in detail reveals heterogeneities on the cellular unit scale^26^. By tightly controlling fluid flows on the chip, we demonstrate the capability of our platform to dynamically regulate the biochemical microenvironment of spheroids and to discriminate changes in the dynamics of cell death upon the application of different drug exposure regimes. Finally, the platform can be extended to the different co-culture configurations, paving the way for new organ-on-chip approaches.

In this study, H4-II-EC3 (a rat hepatoma continuous cell line) is used as a model of liver cells. While these cells do not express all detoxification enzymes of primary hepatocytes, H4-II-EC3 and their derivatives express several important cytochromes (CYP2B1/2, CYP2E, and CYP3A)^27, 28^. In addition, H4-II-EC3 show similar sensibility to pharmaceutical compounds as HEPG2, a well established human hepatoma, widely used for toxicology studies^29^. Moreover, we show that the platform is also compatible with primary progenitor cells, which have broad application in tissue engineering.

## Results

### Droplet-microfluidic platform for the culture of spheroids

**Fig. 1:**
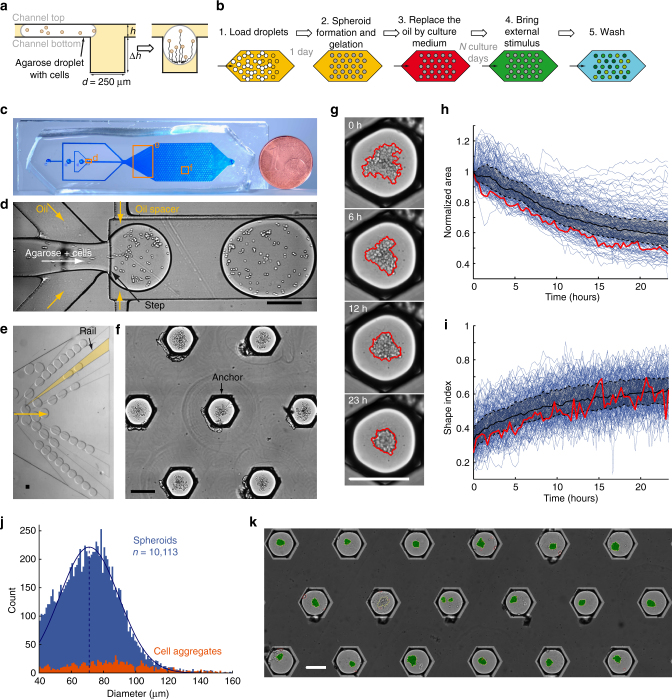
Chip design and protocol. **a** Schematic side view of a droplet entering into an anchor where it remains trapped due to the reduced surface energy. After stopping the oil flow, the encapsulated cells sediment to the bottom of the droplet and form a spheroid after one day of culture (*h* = 95 μm, Δ*h* = 250 μm, and *d* = 250 μm). **b** The protocol begins by loading the drops then gelling them once the spheroids are formed. This allows the oil to be replaced by culture medium in which stimuli or antibodies can be injected. **c** Photograph of the PDMS microfluidic chip. **d** Droplet formation is performed at a flow focusing junction coupled with a step. **e** Diverging rails distribute the droplets over the width of the culture chamber. **f** A few representative anchors with liquid agarose droplets showing the settled cells before spheroid formation. **g** Time lapse of spheroid formation in one anchor. The *red line* shows the edge of the detected pattern. **h**, **i** Dynamics of the spheroid formation: normalized area **h** and shape index **i** evolution (*n* = 152 spheroids). Each *blue line* represents one spheroid. *Red solid line*: spheroid shown in **g**, *black solid line*: median, *black dotted line*: first and third quartiles. **j** Size distribution of all spheroids (*blue*, 35 chips, *n* = 10,113) and cell aggregates (*orange*, *n* = 1,189). **k** Image analysis on 18 anchors filled with spheroids stained for LIVE/DEAD. All *scale bars* are 200 μm

The protocol for spheroid formation and culture is shown schematically in Fig. 1a, b, and takes place in the device pictured in Fig. 1c and Supplementary Fig. 1. The device operation is based on modulating droplet confinement, by working in microfluidic channels that integrate different depths^30, 31^. Since the droplets are attracted to regions where they reduce their confinement, they can be guided and trapped by grooves and holes, patterned in the device surface, which are called “rails” (Fig. 1e) and “anchors” (Fig. 1f)^22^. In the present experiments the anchor dimensions and their hexagonal shape are designed to give each drop a nearly spherical shape when it is trapped. The anchors are placed one droplet diameter apart (680 µm), which yields a density of 500 anchored droplets in 2 cm^2^.

The experiment begins by producing aqueous droplets in fluorinated oil at a flow-focusing junction coupled with a step^32^ (Fig. 1d). The drops have a volume of 16 nL each and contain about 200 cells per droplet, suspended in culture medium mixed with agarose. The droplet production junction is immediately followed by a long “emulsification channel”, in which oil plugs keep the drops well separated, allowing the surfactant adsorption at the water–oil interface^33^. The droplets are then transported to the central chamber, past diverging rails that distribute them across the width (Fig. 1e, Supplementary Movies 1, 2). In the chamber, they are captured by the pre-disposed anchors for the duration of the experiment (Fig. 1f). This loading protocol is realized in 3–5 min and a total of about 800 drops is required to fill the 500 anchors, as some drops exit the chamber without being trapped.

At this stage, the flow of the surrounding oil is stopped and the device is placed in an incubator for 23 h, to allow the cells to settle at the bottom of each drop and form the spheroids (Fig. 1g). The kinetics of spheroid formation of H4-II-EC3 cells in the immobilized droplets can also be examined by live imaging (Supplementary movie 3). For each time point, the spheroid area (*A*) and a shape index (*ShI*), which quantifies the circularity and ranges from 0 (for a straight line) to 1 (for a perfect circle), are calculated in every anchor (Fig. 1h, i). These measurements yield a characteristic time for spheroid formation of about 11 h (Supplementary Fig. 2).

The final shape and diameter (*D*) of the cell structures serve to distinguish cell units (*D* < 40 µm) from spheroids (*D* > 40 µm, *ShI > *0.5) and cell aggregates (*D* > 40 µm, *ShI < *0.5). Cell clusters with *D* < 40 μm are composed of too few cells (about 3–4 cells) to be considered as spheroids. In turn, for *D* > 40 μm the cell aggregates (*ShI < *0.5), which corresponded to poorly interacting cells, only constituted a small part of the population (about 10%) (Fig. 1j). This allowed us to concentrate on well-formed spheroids containing a sufficient number of cells. The average diameter of the spheroids, obtained on more than 10,000 analyzed drops, is 72.9 ± 18.6 μm where the ± indicates the standard deviation (c.v. 25.6%) (Fig. 1j). This variability is mostly due to cell aggregation prior to droplet formation and can be decreased by decreasing the cell concentration. Indeed, using larger drops with the same number of cells reduces the initial cell clustering and results in a tighter size distribution (c.v. 12.4%) (Supplementary Fig. 3).

The effect of spheroid size on hepatocyte viability and function has been extensively investigated in previous studies. It has been demonstrated that limitations of small molecules diffusion could be avoided, while the hepatocyte viability and functions were enhanced, when the spheroid size was maintained around 100 μm^34^. Consequently, the potential interference of nutrient and oxygen diffusion is not expected to have any significant influence in the current measurements of the cells’ behavior. Moreover, this spheroid size range allows the diffusion of antibodies, which facilitates the accurate quantification of the cells’ biological properties within the 3D structures^35, 36^.

A critical step is then performed after the complete process of spheroid formation: the device is placed at 4 °C for 30 min in order to solidify the agarose droplets. This gelation allows the surrounding oil to be replaced by an aqueous medium (Supplementary Movie 4), since the gel beads are now trapped by their mechanical resistance rather than by the water–oil interfacial tension. This switching of the external phase allows the spheroid culture for several days, by providing a way to bring fresh culture medium to the cells. It also allows water-soluble molecules to be injected into the culture chamber, where they diffuse into the gel beads in order to stimulate or stain the cells. The spheroids remain firmly anchored in place during all of the medium changes and washing steps, even in the presence of flows (Supplementary Fig. 4), in contrast with typical protocols in which changing media implies the spheroid displacement. Finally, fluorescence and bright-field images are acquired, using a motorized microscope, and are analyzed by our in-house software to provide a global and detailed view of the chip contents (Fig. 1k and Supplementary Figs. 5, 6).

### Multiscale cytometry on chip

**Fig. 2:**
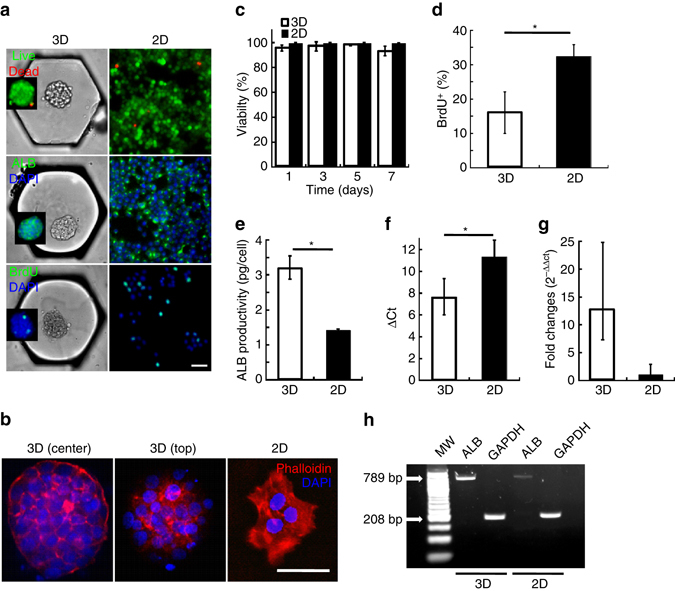
Population-level comparison of 3D vs. 2D cultures. **a** Representative images of spheroids in the anchors and 2D cells stained for LIVE/DEAD, ALB and BrdU. **b** Actin organization in one spheroid on chip (confocal slices at two vertical positions), and cells in 2D. **c** Evolution of the viability of the spheroids on chip and of the cells in 2D, during a 7-day culture period (*n*_chips_ = 3; *n*_2D_ = 4). **d** Quantification of the percentage of BrdU^+^ cells in spheroids on chip and cells in 2D, over a 7-day culture period (*n*_chips_ = 5; *n*_2D_ = 4). **e** ALB productivity at day 4 by spheroids on chip and cells in 2D, measured by ELISA on the supernatant (*n*_chips_ = 2; *n*_2D_ = 3). **f** RT-qPCR analysis of relative ALB expression to GADPH (ΔCt), in 3D and in 2D (*n*_chips_ = 3; *n*_2D_ = 3) **g** RT-qPCR analysis of ALB expression (Relative RNA expression), in 3D and in 2D (*n*_chips_ = 3; *n*_2D_ = 3). **h** Representative gel of RT-PCR analysis of ALB and GAPDH expression, in 3D and in 2D. All *scale bars* are 50 μm. *Error bars* show the standard deviation of each population. *p < 0.05, unpaired two-sample two-tailed Student’s *t*-tests

The spheroids were first analyzed on the scale of the whole population, making use of the compatibility of the platform with standard protocols. Cytochemistry was used to assess the viability (LIVE/DEAD staining), actin organization (phalloidin-Alexa594 staining), proliferation (BrdU staining), and functionality of the cells, using albumin (ALB) as a marker of hepatocyte function^37^ (Fig. 2a). All measurements were compared between the 3D and standard 2D cultures, revealing significant differences between the two configurations (Fig. 2b, d–f). We first note that the F-actin staining revealed distinct cytoskeletal organization between the spheroids and the 2D cultures, with the cells within the spheroids showing mainly cortical actin, compared with the dominance of cytoplasmic fibers for F-actin in 2D cells (Fig. 2b).

During the 7 culture days, a similar cell viability between the spheroids and the 2D cultures (above 95%) was measured by image analysis (Fig. 2c), while a 2-fold decrease in the percentage of BrdU positive cells was observed in 3D (about 16%) versus the monolayers (almost 30%) (Fig. 2d). The ALB production was quantified off-chip, first by ELISA on the culture supernatant, then by reverse transcription (RT^−^)qPCR on the extracted spheroids. At the protein level, the results showed a doubling of ALB production between 3D (3 pg per cell) and 2D cultures (1.5 pg per cell), after a 4-day culture period (Fig. 2e). At the transcriptional level, an almost 10-fold increase in RNA expression was measured in the spheroids as compared to the cells in 2D (Fig. 2f–h). The alteration of cytoskeletal organization, the reduced proliferation and the increased ALB expression compared to 2D demonstrated that H4-II-EC3 cultivated on the microfluidic platform bear the structural and phenotypic features of highly functional hepatocyte spheroids^38, 39^.

**Fig. 3:**
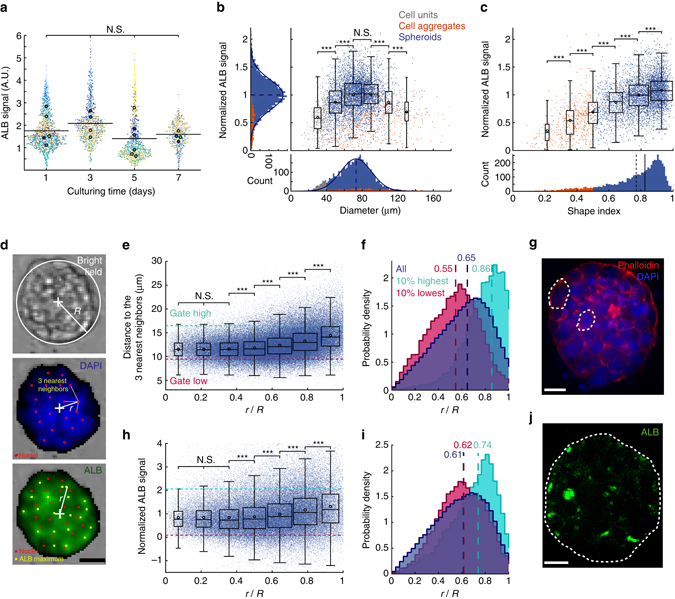
Multiscale cytometry on chip. **a** Measured amplitude of intracellular ALB. Each *dot* represents one spheroid and each color represents one chip. The mean value for each chip is shown by a *larger dot* with the same color. *Solid black line*: average ALB signal at each time point. D + 1/D + 3/D + 5/D + 7: *n* = 5/4/6/4 chips; *N* = 4,925 spheroids. **b**, **c** Normalized ALB signal for each spheroid vs. **b** the spheroid diameter and **c** the shape index. The normalization was performed by dividing a specific spheroid ALB intensity value by the average ALB signal within a chip. By this way, it was possible to compare ALB production of spheroids from different chips. The histograms show the distribution of the data along the different axes. *Blue curves* show Gaussian fits, *dotted blue line*: mean, *solid blue line*: median. *N*_spheroids = _4,925, *N*_cell aggregates = _506, and *N*_units_ = 389. **d** Representative images for a spheroid in bright field (top), stained for DAPI (middle), and for ALB (bottom). *R* represents the effective spheroid radius and *r* the radial coordinates of each detected peak in the fluorescent signal within the spheroid. *Scale bar* is 50 μm. **e** Mean distance between each cell and its 3 nearest neighbors (*n*_cells_ = 128,973, *N*_spheroids_ = 6,236). *dotted magenta line*: first decile and *dotted cyan line*: last decile of the distances. **f** Corresponding histograms of all the nuclei and of the two gated populations of part **e**. (*magenta*=below the first decile; *cyan*=above the last decile). *Dashed lines* give the median values. **g** Confocal image of the mid-plane of a spheroid stained for phalloidin (*red*) and DAPI (*blue*). *Dashed lines* emphasize two representative cells. **h** Normalized ALB vs. distance from spheroid center (*n*_ALB maxima = _87,609, *N*_spheroids_ = 4,654). **i** Corresponding histogram for gated populations. **j** Confocal image of the mid-plane of a spheroid stained for ALB (*green*). *White dashed line* shows the spheroid edge. Non significant N.S., ***p < 0.001. **a**, **b** Kruskal–Wallis ANOVA followed by Mann–Whitney *U*-tests with Sidak’s correction for multiple comparisons. **c** Welch’s ANOVA followed by Games-Howell post hoc procedure

A deeper level of detail could be obtained by studying the data at the scale of each spheroid and by quantifying variations in shape and function within the population. Indeed, although the spheroid population (about 5,000 spheroids) did not show any significant evolution in intra-cellular ALB signal with culture time (Fig. 3a), individual spheroids within each experimental run displayed variations related to their size and shape. The intracellular ALB expression, obtained by immunocytochemistry (ICC), correlated with the diameter and displayed a maximum value for spheroids of diameter in the range of 60–100 μm (Fig. 3b, Supplementary Fig. 7a for individual runs)^34^. More significantly, the intracellular ALB signal correlated strongly with the shape index and displayed a continuous three-fold increase when *ShI* increased from 0.2 to 1 (Fig. 3c, Supplementary Fig. 7b for individual runs).

The effect of *ShI* on ALB production was further confirmed by prematurely solidifying the agarose, before the cells were able to form circular spheroids (Supplementary Fig. 8a, b). Blocking the spheroid formation at *t* = 3 h reduced the *ShI* by 1.5-fold and led to a 2-fold decrease of the ALB signal compared to the gelation at *t* = 20 h (Supplementary Fig. 8c, d, e, f). In turn, the agarose gelation at *t* = 6 h resulted in a mixed spheroid population displaying a broad range of *ShI* values. The spheroids with low *ShI* (0.2–0.5) showed low ALB signal, while those with higher *ShI* (0.8–1) expressed higher intracellular ALB (Supplementary Fig. 8c, d, e, f). This dependence of productivity on shape supported the role of functional cell–cell interactions to promote the spheroid compaction and ALB expression^40–42^.

Finally, measurements at the scale of individual cells were performed, which yielded high-throughput, quantitative information on the cell behavior in situ within each spheroid. Detailed information on the 3D organization was obtained by a combined analysis of bright field and fluorescent images (Fig. 3d) and confirmed using confocal imaging on a few representative examples. First, the analysis of the DAPI staining (130,000 nuclei from 6,000 spheroids) evidenced a distinct cellular organization in the core versus the edge of the spheroids. For instance, the mean distance between neighboring nuclei was found to be larger near the edge compared with the core of the spheroid (Fig. 3e, f). This quantitative trend was supported by confocal images in which the nuclei in the core were observed to be more compact and denser compared with the edge of the spheroids (Fig. 3g). Indeed, the cells in the interior of the spheroids were round, while some flat and enlarged cells were observed on the top of the 3D structure (Fig. 3g), a structural characteristic of well organized spheroids of hepatocytes^43, 44^.

From a functional point of view, an analysis on 5,000 spheroids indicated that the intensity of ALB peaks correlated with the cell location within the 3D structure (Fig. 3h). A continuous increase in signal intensity of ALB was observed from *r*/*R* = 0.4 to 1 (where *r*/*R* is the normalized distance to the spheroid center), while a lower and flat signal was measured between *r*/*R = *0 to 0.4 (Fig. 3h, i). The signal from individual experiments showed a similar trend (Supplementary Fig. 7c), and the 3D confocal images indeed displayed more intense ALB signal near the edge of the spheroids (Fig. 3j). This increase of ALB with radial position may be a consequence of the mechanical properties of the agarose hydrogel, and is consistent with previous observations^45^. Indeed, while ALB distribution is homogeneous within spheroids cultivated in free suspended cultures^46^, recent investigations suggest that the encapsulation of hepatoma spheroids significantly reduces ALB in the center, due to the capsule rigidity^45^.

In the same vein, the fluorescent signal analysis demonstrated a slightly higher percentage of proliferative cells (BrdU^+^) between *r*/*R = *0.8–1, compared to the spheroid center between *r*/*R = *0–0.5 (Supplementary Fig. 9). Although some proliferating cells were detected, the spheroid diameters remained stable along the 7-day culture period (Supplementary Fig. 10). Similar trends were observed for other types of spheroids encapsulated in agarose^47^ and it was postulated that potential solid stress generated in the hydrogel impaired the cell proliferation at the interior of the spheroid^44, 47^, since diffusion limitation was unlikely to occur for these sizes. Indeed, low molecular weight compounds (e.g., fluorescein) were able to diffuse homogeneously and rapidly (i.e., within few minutes) inside the agarose beads and the spheroids.

Finally, the same quantitative methodology was also applied for the detection and the spatial distribution of the dead cells within 4000 spheroids. The results revealed that most of the dead cells were located near the edge of the spheroids, at the interface with the agarose, while no necrotic region was observed in the core of the spheroids (Supplementary Fig. 11). The dead cells that were detected at the edges of the spheroids may have been ejected from the spheroids due to their inability to establish cell–cell interactions^41^. Alternatively, they may simply have been dead before the spheroid formation step.

Similar observations on human mesenchymal stem cells (hMSCs) (Supplementary Fig. 12), show that the platform is compatible with the formation and the culture of viable (viability was 96%) and well-organized spheroids derived from primary cells. The same cytometry approach as above shows quantitative and qualitative differences between the hMSCs and H4-II-EC3 in our platform. First, the characteristic time for the formation of hMSC spheroids is significantly shorter than for hepatoma (*τ* = 4 h vs. 11 h) (Supplementary Fig. 12a, b, c). Second, most of the dead cells are found in the core of the spheroids of hMSCs, in contrast with H4-II-EC3 where the dead cells are mostly on the edges (Supplementary Fig. 12d–h).

Together, the above results demonstrate the capability of our device to support cell proliferation and phenotype preservation under long-term static culture. In the following sections of this work, we demonstrate three other unique features of the platform, in which the cytometry capabilities can be combined with other microfluidic methods, namely (i) the dynamic monitoring of individual cell behavior within the spheroids under controlled perfusion, (ii) the spatially and temporally controlled co-culture of cells in 3D and 2D configurations, and (iii) the selective recovery of an individual spheroid.

### Multiscale cytometry under dynamic drug perfusion

**Fig. 4:**
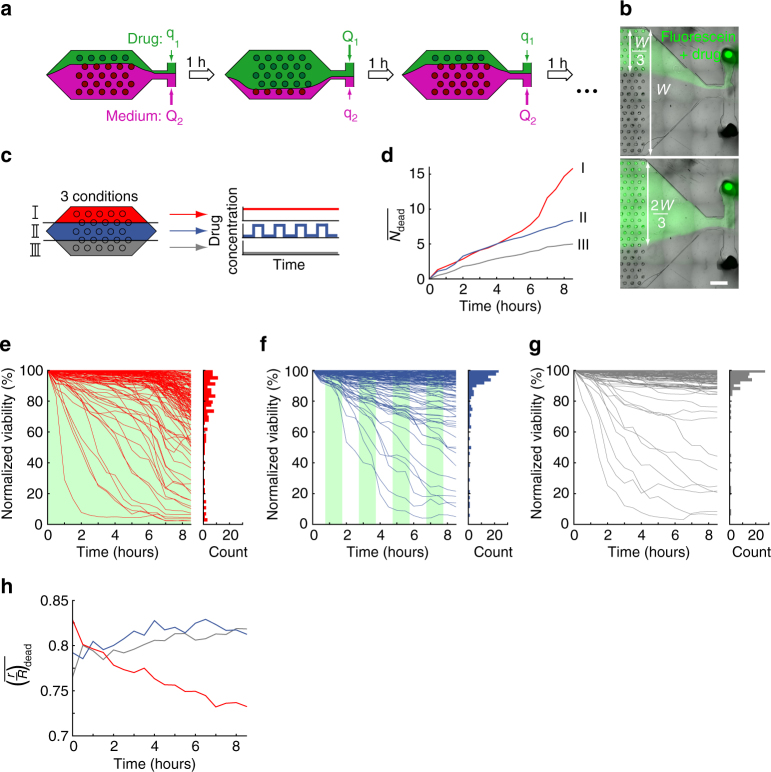
Multiscale cytometry under dynamically regulated drug perfusion. **a** The chamber is perfused with two solutions: one contains only culture medium, while the other is supplemented with 300 μM acetaminophen. The control of the relative flow rates enables to regulate spatially and temporally the spheroids’ exposure to the drug. **b** Micrograph of the chip perfusion with different solutions. Fluorescein marks the solution containing the drug. *W* is the width of the chamber. *Scale bar* is 1 mm. **c** H4-II-EC3 spheroids are exposed to three different culture conditions in the same chamber. The top part (region I) of the chip is continuously perfused with the drug (*red*). The middle part (region II) is intermittently perfused with the drug, with a half-period of 1 h (*blue*). The bottom of the chamber (region III) is perfused with culture medium without drug (*gray*). **d** Time evolution of the mean number of dead cells, normalized by the number of spheroids in each region ($\overline {{N_{{\rm{dead}}}}}$), in the 3 different regions (I=*red*, II=*blue*, III=*grey*). **e**–**g** Time evolution of the viability for each spheroid in the 3 experimental conditions: **e** region III (*n* = 131 spheroids), **f** region II, (*n* = 133 spheroids), **g** region I (*n* = 106 spheroids). The *green* background indicates the drug perfusion. The histograms indicate the viability distribution at the end of the experiment for each condition. **h** Time evolution of the mean normalized distance of the dead cells to the spheroid center, ($\overline {{{\left( {\frac{r}{R}} \right)}_{{\rm{dead}}}}}$) in the three different regions

In this experiment, 500 spheroids were formed on the microfluidic chip and the oil was replaced with an aqueous medium, as above. However, the chip design was slightly modified to allow two streams to be pushed into the culture chamber after the spheroid formation (Fig. 4a); one of the streams was connected to pure culture medium, while the other was connected to a solution marked with fluorescein and supplemented with acetaminophen, a drug known to be toxic for hepatocytes (Fig. 4b)^48^. This design could be extended towards the perfusion of specific lines of spheroids (Supplementary Fig. 13). Three conditions were explored on the chip (Fig. 4c): in the top zone (region I), the spheroids were subjected to the drug for the whole duration of the experiment. In the bottom zone (region III), the spheroids were never subjected to the drug. Finally, in the middle zone (region II), the spheroids were subjected to the drug intermittently, with a half-period of 1 h.

If the experiments were conducted on current state of the art equipment, one would measure the cell viability as a function of the incubation time with the drug. The equivalent measurement was made continuously in our device, without the need to sample the cells off-chip. This population-scale measurement showed that the number of dead cells increased in all three regions, but with marked differences (Fig. 4d). The negative control (region III) showed limited cell death, increasing at a slow but constant rate (*gray line*). The intermittently drug-exposed population (region II) showed a faster increase (2-fold) in the number of dead cells (*blue line*). The mortality of the cells in the continuously exposed region (region I) closely matched that of region II, up to a time of about 5 h, after which a sharp increase (4-fold) was observed (*red line*). Importantly, the flow rate in region II was constant and maintained at 40 μL min^−1^ for the duration of the experiment, as in the regions I and III. As a consequence, it is unlikely that flow switching caused cell death in region II.

Looking at the data on the spheroid scale provides a much more complete picture of the cell death kinetics (Fig. 4e–g). The vast majority of the spheroids cultivated in the absence of the drug showed high viability (region III). The cell deaths that were detected on the population scale were concentrated in a small number of spheroids (10 out of 106) that showed a decreased viability in the first hours in culture (Fig. 4g). A similar behavior was observed in region II, though the number of spheroids that died was higher (28 out of 133 spheroids) (Fig. 4f). In both of these regions, this led to a final distribution dominated by mostly high-viability spheroids with some low-viability individual cultures. In contrast, the viability in region I showed very different dynamics. First, a similar rapid decrease in spheroid viability was observed for 25 out of 131 spheroids. But after 6 h of incubation with the drug, a generalized decrease in viability was observed for 53 out of 131 spheroids (Fig. 4e). The final distribution of viability thus displayed a much lower value and a wider spread compared with the two previous conditions.

The dual kinetics of cell death in region I suggest that two different mechanisms take place under continuous exposure to the drug, one of which is fast and the other slow. The cytotoxicity of acetaminophen is induced by the formation of N-acetylimidoquinone (NAPQI) and gradual glutathione (GSH) depletion^48, 49^. H4-II-EC3 produce the several cytochromes involved in the NAPQI production^50^. Our observations are consistent with the known mechanisms of the toxicity induced by acetaminophen, which occurs through an initial induction of oncotic necrosis (rapid induction promoted by NAPQI) followed by apoptosis (longer induction, requiring GSH depletion)^48^. In the intermittent zone (region II), only the fast mechanism is observed while the slow mechanism is not reached. This result suggests that in the region II, NAPQI does not accumulate (or may be washed out when the flows are switched) and GSH remains at a sufficient level to promote cell survival.

The contrast between the three conditions was also visible at the cellular level, as shown in Fig. 4h, where the mean position of the dead cells within the spheroids was plotted as a function of time. In regions II and III, cell death was observed near the boundaries of the spheroids (*r*/*R = *0.8), and remained stationary for the duration of the experiment. In contrast, the location of the dead cells in region I started near the same position (*r*/*R* = 0.8) but gradually shifted inward (Fig. 4h). This indicated an increase in the occurrence of cell deaths in the inner parts of the spheroids.

Again, these results are consistent with the different mechanisms of cell death induced by acetaminophen^48^. These mechanisms are expected to correlate with the cell proliferation and energetic status^36, 48^, which in turn vary with the position of the cells within the spheroid (Supplementary Fig. 9). Moreover, our results suggest that continuous acetaminophen exposure is required to induce apoptosis, which can be due to the time required for acetaminophen to induce mitochondrial pore opening^51^.

### Towards 3D and mixed 3D–2D organs-on-a-chip

The methods shown above can naturally be extended to co-culture systems and organ-on-a-chip techniques. This can be done either by mixing together different cell types within each droplet, thus obtaining a 3D organoid in each anchor, or by considering the whole chip as a single organ, for instance by mixing 3D and 2D cultures. We demonstrate these two extensions by co-cultivating bovine aortic endothelial cells (BAECs) with the H4-II-EC3 spheroids.

**Fig. 5:**
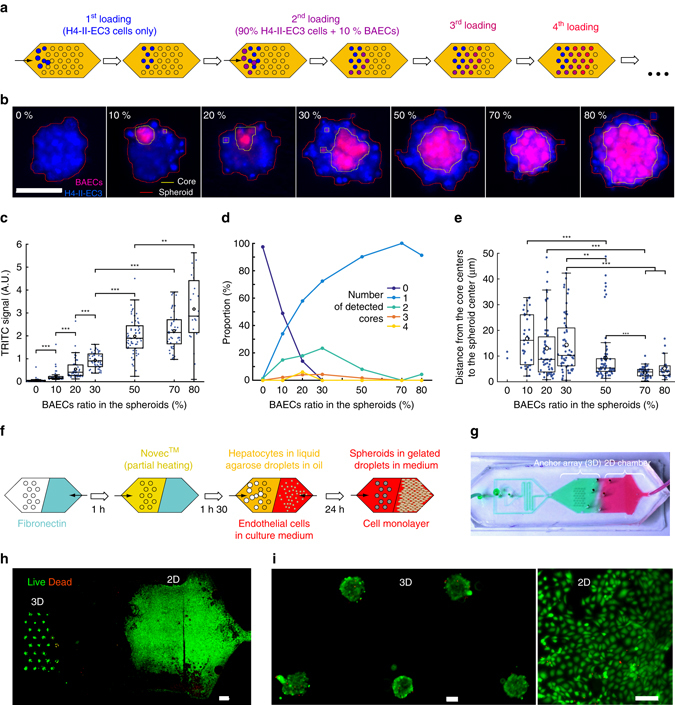
Co-culture of endothelial cells (BAECs) and 3D hepatoma (H4-II-EC3) in the 3D spheroids **a**–**e** and in mixed 3D–2D conditions **f**–**i**. **a** Schematic diagram of the loading protocol for having different conditions on a single droplet array. 7 cell solutions with an increasing ratio of BAECs were successively loaded. **b** Fluorescent images of one representative spheroid for each BAECs ratio. The BAECs are stained with CellTracker Red and all cells with NucBlue Live reagent. The *red* and *yellow lines* represent respectively the edges of the detected spheroids and BAECs cores. On these representative images the contrast is individually set on each frame. *Scale bar* is 100 μm. **c** Variation, as a function of the BAECs cell ratio, of **c** TRITC signal **d** the number of detected BAECs cores, and **e** the distance of the core centers to the spheroid center (1 chip, N_spheroids_ = 355, N_cores_ = 292). **f** Schematic diagram of the mixed 3D–2D protocol: the region for 2D culture is coated with fibronectin, the region for spheroid culture is coated with Novec. After differential surface treatment, cells are seeded in the anchors within droplets for spheroid formation, or directly on PDMS treated with fibronectin for 2D culture. **g** The chip design enables the spatial control over the deposition of different coating solutions, while preventing their mixing. **h** LIVE/DEAD stained hepatoma spheroids and BAECs monolayer demonstrate controlled spatial distribution. *Scale bar* is 1 mm. **i** Higher magnification of the spheroids and BAECs in 2D, stained for LIVE/DEAD. *Scale bars* are 100 μm. **p < 0.01, ***p < 0.001. Kruskal–Wallis ANOVA followed by Mann–Whitney *U*-tests with Sidak’s correction for multiple comparisons

The simplest co-culture model is the direct interaction between two different cell types in 3D. To interrogate the influence of the different cell populations on the structural organization of the hetero-spheroids, several ratios of BAECs and H4-II-ECs were mixed together. The use of droplets to transport and form the spheroids allowed us to have all of the different conditions on the same chip, by sequentially forming droplets that contained the different mixing ratios (Fig. 5a). In each anchor, the cells were found to self-organize into a single spheroid, as shown in Fig. 5b, with the BAECs being located in the inner part of the spheroids and surrounded by H4-II-EC3, whatever the cell count ratio. The protocol resulted in 7 independent co-culture conditions and 50 replicates for each condition on a single chip (Fig. 5c).

These observations contrasted with previously reported measurements where the spatial organization of the co-culture depended on the relative abundance of different cell types^52^. Instead we found that at small cell ratios (10–50% of BAECs) the hetero-spheroids contained several cores of BAECs, while the higher ratio conditions (70–80% of BAECs) showed a single well-defined cluster of endothelial cells (Fig. 5d). Moreover, the location of the cores of BAECs depended on the relative abundance of the two different cells types: the cores formed with high BAECs percentages were more likely to be centered than the cores made with smaller BAECs percentages, which were distributed farther away from the spheroid center (see Fig. 5e).

It should be noted however that the cell self-organization begins during the spheroid formation, as the cells start to interact together, but that it can continue over long periods, driven by cell motility within the spheroids^53^. We therefore expect that the current observations for the low BAECs concentrations correspond to metastable situations that can evolve with time, as the different small cores meet and fuse together in a ripening process.

In addition to 3D co-cultures, many organ-on-a-chip platforms provide the ability to co-culture 2D monolayers in order to take advantage of paracrine signaling between the cells^54^. However none allow the co-culture of 3D spheroids with monolayers, which would be a relevant model for such organs as the liver, where the 3D plates of hepatocytes are physically separated from the layer of liver sinusoidal endothelial cells by the space of Disse^55^, but with which they interact through paracrine signaling. For instance, angiocrine factors play significant roles in the homeostasis, the regeneration and the induction of pathologic events in the liver^56^. Hence, to investigate the effects of paracrine signaling, the two cell types need to be physically segregated^57^ and cultivated under different conditions to support their specific metabolic activities^58^.

Such situations can be obtained in the current platform, by taking advantage of the ability to place droplets at pre-defined locations. Therefore by combining the patterning of the anchors with a differential coating of the different channel regions (Fig. 5f, g), it is possible to obtain a co-culture system that combines 2D and 3D cultures with a separate spatial and temporal control of the different cell types. In the current demonstration, the droplets containing the hepatoma cells are first loaded and anchored on the left side of the chip. After the spheroid formation, the BAECs are injected and they spread on the PDMS (Fig. 5f). Both cell populations were viable after 48 h in culture (Fig. 5h, i). Importantly, this new culture configuration is totally compatible with the “multilevel cytometry” analysis and perfusion system, described above.

The devices shown here can serve as building blocks to test a very wide range of spatial and temporal organization that can be tailored depending on the specific questions to be addressed.

### Selecting desired spheroids

**Fig. 6:**
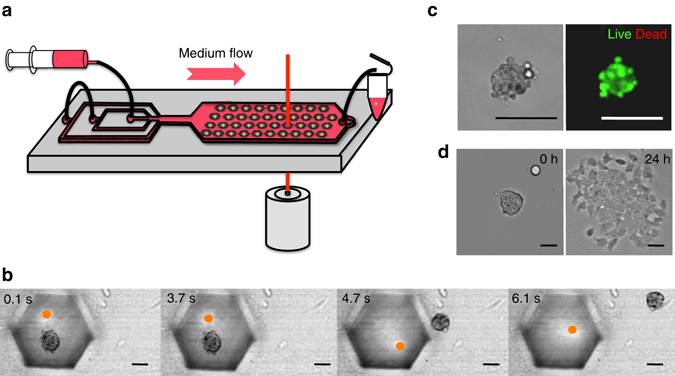
Selective spheroid recovery. **a** Schematic protocol: the selected agarose droplet is locally heated with a focused infrared laser beam (*orange d﻿ots*). A culture medium flow transports the spheroid out of the chip, where it is collected in a 1.5 mL tube. **b** Time lapse of spheroid recovery. *Orange dots* indicate the laser position in the anchor. **c** Bright field and LIVE/DEAD stained spheroid after laser recovery. **d** Bright field picture of a spheroid immediately after replating (0 h) and 24 h post-replating in 2D dish. All *scale bars* are 50 μm

An important feature is the ability to sort and recover cells of interest. In addition to the culture and observation capabilities, the current platform enables the selective recovery of a spheroid by implementing the protocol sketched in Fig. 6a. Here, a focused infrared laser is used to melt the desired agarose bead by localized heating^59^, while a flow of culture medium is used to carry the detached spheroid out of the microfluidic chip (Supplementary Movie 5, Fig. 6b). After the laser sorting, the spheroids are still viable (Fig. 6c) and the cells maintain their capability to migrate out of the 3D structure when replated on 2D dishes (Fig. 6d).

## Discussion

This study demonstrates the integration of several technologies into a single microfluidic platform for 3D cell culture, dynamic regulation, “multiscale cytometry” (i.e., population, spheroid, single cell levels), quantitative image analysis, co-cultures and selective recovery. The same cells can be observed from seconds to days, without any modification of the protocols, and any spheroid can be extracted at any point. As a proof of concept, the robustness of the platform was demonstrated by the preservation the cells’ biological properties in 3D while providing a level of statistical robustness and accuracy that significantly exceed those presented in the current literature.

The present quantitative analysis of over 10,000 individual spheroids and 130,000 cells, in situ within their microenvironment, provides a set of data that is comparable in throughput to flow cytometry. The platform thus provides a window to observe cell heterogeneity within their specific niche, by giving access simultaneously to structural and functional information^8, 9^. However, in contrast with other platforms, the microfluidic approach provides a simple way to dynamically regulate the culture environment or to spatially structure the 3D and 2D cultures on the same chip. When combined with our analysis method, the platform provides access to subtle kinetic trends, at the single cell level, which could not have been achieved by any other method.

The droplet-based approach also allows the current platform to be combined with existing droplet tools. Indeed, the next developments will consist in expanding the droplet level operations to allow each anchored spheroid on the chip to be exposed to a different stimulus. This can be achieved for example by introducing a library of droplets, e.g., containing different drugs and different concentrations, into the chip and merging them with the anchored droplets containing the spheroids. Such libraries can contain reagents acting on the cells or they can react with the gel in order to modify the physical or chemical properties in each drop.

Finally, the microfluidic platform can be integrated into high-content screening systems (e.g., ImageXpress) for an increased throughput in the analysis of cell behavior in 3D. The quantitative capabilities of such an integration have the potential to transform cellular assays in the context of organs-on-chips^54^, stem cell differentiation^25, 60^, or drug screening^61^.

## Methods

### Microfabrication

Standard dry film soft lithography was used for the flow focusing device (top of the chip) fabrication, while a specific method for the fabrication of the anchors (bottom of the chip) was developed. For the first part, four layers of dry film photoresist consisting of 33 μm Eternal Laminar E8013 (Eternal Materials, Taiwan) and 15 μm Alpho NIT215 (Nichigo-Morton, Japan) negative films were successively laminated using an office laminator (PEAK pro PS320) at a temperature of 100 °C until the desired channel height, *h*_0_ = 95 μm was reached. The photoresist film was then exposed to UV (LightningCure, Hamamatsu, Japan) through a photomask of the junction, channels and the culture chamber boundaries. The masters were revealed after washing in a 1% (w/w) K_2_CO_3_ solution (Sigma-Aldrich). For the anchors fabrication, the molds were designed with RhinoCAM software (MecSoft Corporation, LA, USA) and were fabricated by micro-milling a brass plate (CNCMini-Mill/GX, Minitech Machinery, Norcross, USA). The topography of the molds and masters were measured using an optical profilometer (VeecoWyco NT1100, Veeco, Mannheim, Germany).

For the fabrication of the top of the chip, poly(dimethylsiloxane) (PDMS, SYLGARD 184, Dow Corning, 1:10 (w/w) ratio of curing agent to bulk material) was poured over the master and cured for 2 h at 70 °C. For the fabrication of bottom of the chip, the molds for the anchors were covered with PDMS. Then, a glass slide was immersed into uncured PDMS, above the anchors. The mold was finally heated on a hot plate at 180 °C for 15 min. The top and the bottom of chip were sealed after plasma treatment (Harrick, Ithaca, USA). The chips were filled three times with Novec Surface Modifier (3 M, Paris, France), a fluoropolymer coating agent, for 30 min at 110 °C on a hot plate.

### Cell culture

A rat H4-II-EC3 hepatoma cell line (CRL-1600, American Type Culture Collection, LGC, Molsheim, France) was maintained on T-25 cm^2^ flasks (Corning, France) in a standard CO_2_ incubator (Binder, Tuttlingen, Germany), following the instructions provided by the manufacturer (ATCC). The culture medium was composed of Dulbecco’s Modified Eagle’s medium (DMEM)-containing high glucose (Gibco, Life Technologies, Saint Aubin, France) supplemented with 10% (v/v) fetal bovine serum (Gibco) and 1% (v/v) penicilin-streptamicine (Gibco). The cells were seeded at 5 × 10^4^ cells cm^−2^ and sub-cultivated every 3 days. No additional ECM protein (i.e., other than those provided by FBS) or sandwich culture was used in this study.

BAECs (B-304-05, Cell Applications, Inc.) were cultivated in T-25 cm^2^ flasks, as above. For sub-cultivation, BAECs were expanded in a Bovine Endothelial Growth medium (Cell Applications, INC., San Diego, USA). Passage 8 to 9 cells were used in the co-culture experiments with H4-II-EC3, for which DMEM-containing serum was used.

Human mesenchymal stem cells derived from the Wharton’s Jelly of umbilical cord (hMSCs) (ATCC PCS-500-010, American Type Culture Collection, LGC, Molsheim, France) were obtained at passage 2. hMSCs were maintained in T-175 cm^2^ flasks (Corning, France) and cultivated in a standard CO_2_ incubator (Binder, Tuttlingen, Germany). The culture medium was composed of Alpha Modified Eagle’s medium (α-MEM) (Gibco, Life Technologies, Saint Aubin, France) supplemented with 10% (v/v) fetal bovine serum (Gibco) and 1% (v/v) penicilin-streptamicine (Gibco). The cells were seeded at 5 × 10^3^ cells cm^−2^, sub-cultivated every week, and the medium was refreshed every 2 days. hMSCs at passage 2 were first expanded until passage 4 (for about 5–6 populations doublings, PDs), then cryopreserved in 90% FBS/10% DMSO and stored in a liquid nitrogen tank. The experiments were carried out with hMSCs at passage 8 (about 24–35 PDs, after passage 2).

### On-chip cell loading, spheroid formation, culture, and recovery

Passage 4–35 cells were collected with trypsin at 60–70% confluence and a solution containing 1.2 × 10^6^ cells in 70 μL medium were mixed with a 30 μL 3% (w/v) liquid low melting agarose solution (i.e., stored at 37 °C) (Sigma-Aldrich, Saint Quentin Fallavier, France) diluted in culture medium (1:3 v/v), resulting in a 100 μL solution of 12 × 10^6^ cells mL^−1^ in 0.9% (w/v) agarose.

Cells and agarose were loaded into a 100 μL glass syringe (SGE, Analytical Science, France), while Fluorinert FC-40 oil (3 M, Paris, France) containing 0.5% (w/w) PEG-di-Krytox surfactant (RAN Biotechnologies, Bervely, USA) was loaded into 1 mL glass syringes (SGE, Analytical Science). Droplets of cell-liquid agarose were generated in the FC-40-containing PEG-di-Krytox, at the flow focusing junction, by controlling the flow rates using syringe pumps (neMESYS Low Pressure Syringe Pump, Cetoni GmbH, Korbussen, Germany). After complete loading, the chips were immersed in PBS and the cells were allowed to settle down and to organize as spheroids for 20 h in the CO_2_ incubator. Then, the agarose was gelled at 4 °C for 30 min, after which the PEG-di-Krytox was extensively washed by flushing pure FC-40 in the culture chamber. After washing, cell culture medium was injected to replace the FC-40. All flow rates are indicated in Supplementary Table 1.

The spheroids were cultured on-chip for 1, 3, 5, and 7 days. Different sterility procedures were used for short and long-term cultures. For culture <3 days, the cell-agarose solution was loaded under the flame of a burner. Alternatively, for culture >3 days, the chips equipped with tubing and connection needles were autoclaved and all of the tubes were connected to syringe filters (Acrodiscs, pore size=0.22 μm, Pall Scientific, Portsmouth, UK) under laminar flow hood after the chip loading.

For selective spheroid recovery, the spheroids were allowed to settle to the top of the chip by turning the chip over before placing at 4 °C. For the extraction, the gelled agarose bead was locally heated with a laser (ILX, light ware; wavelength *λ* = 1480 nm, power *p* = 50 mW)^59^, while culture medium was flushed at 100 μL min^−1^. The spheroids were then recovered in a 1.5 mL tube prior to analysis or replating into a 24-well plate. For the global recovery of all spheroids, the agarose beads were gently mechanically disturbed under a 500 μL min^−1^ flow, and the spheroids were collected in 1.5 mL tube, as above.

### Live analysis of spheroid formation

For the live imaging of the spheroid formation, the chips were immersed in PBS, and then were incubated for 24 h in a microscope incubator equipped with temperature, CO_2_ and hygrometry controllers (Okolab, Pozzuoli, Italy). The cells were imaged every 20 min.

### Viability assay

The cell viability was assessed using LIVE/DEAD staining kit (Molecular Probes, Life Technologies). The spheroids were incubated for 30 min in PBS containing 1 μM calcein AM and 2 μM ethidium homodimer-1 (EthD1), in flushing 100 μL of the solution. The samples were then washed with PBS and imaged under a motorized fluorescent microscope (Nikon, France).

### Enzyme-linked immunosorbent assay for quantification of secreted albumin

The culture supernatants of T-25 cm^2^ flasks were collected, while the total medium content of the chip was recovered by flushing the culture chamber with pure oil. Next, the whole cell population was recovered from the chip, and the culture medium was separated from oil by their difference in density. The total volume of culture medium was measured. Then, ALB concentration was evaluated using a standard ELISA kit, following the manufacturer instruction. Total ALB proteins weight in the supernatant was calculated by multiplying the measured ALB concentration by the total collected volume. Then, ALB weight was normalized by the total cell number in the chip.

An ALB rat ELISA kit (ab108789, Abcam, Cambridge, UK) was used for the ALB quantification, following the manufacturer instructions. Briefly, a standard curve of ALB concentration derived from the serial dilution of an ALB standard solution was generated (*r* ^2^ > 0.9). The absorbance was measured using a plate reader (Chameleon, Hidex, Finland). The ALB productivity was calculated by normalizing the total amount of secreted ALB by the total number of cells contained in the T-flasks or harvested from the chips. Culture medium was used as a negative control. The absence of signal for the negative control indicated the signal specificity to the ALB produced by the cells.

### Detection of intracellular albumin expression

The intracellular ALB production was investigated by ICC, for which all steps were carried out on-chip. The cells cultivated on T-25 flasks or as spheroids on-chip were fixed in a 4% (w/v) PFA (Alpha Aesar, Heysham, UK) for 30 min and permeabilized with 0.2–0.5% (v/v) Triton X-100 (Sigma-Aldrich) for 5 min. The samples were blocked with 5% (v/v) FBS in PBS for 30 min and incubated with sheep polyclonal anti-rat ALB antibody conjugated with FITC solution (ab93322, Abcam, Cambridge, UK) diluted at 1:200 in 1% (v/v) FBS for 30 min. The cells were counterstained with 0.2 μM DAPI for 5 min (Sigma-Aldrich), and then washed with PBS. To ensure the specificity of the antibody to the cells’ secreted ALB, control cells were permeablized and incubated with a sheep IgG Alexa Fluor 488 isotype (IC016G, R&D systems, Lille, France), following the manufacturer instructions. To verify the absence of contribution of the ALB from the blocking buffer in the fluorescent signal, the cells were treated as above and co-incubated with the anti-rat ALB antibody and 10 μg mL^−1^ BSA-tetramethylrhodamine conjugate (Life Technologies) for 30 min. The samples were imaged using fluorescent microscope or using confocal microscopy. The absence of fluorescence for the isotype and the BSA-tetramethylrhodamine controls indicated the signal specificity to the cells’ produced ALB.

### BrdU staining

To assess the fraction of proliferative cells within the spheroids or in 2D cultures, the samples were incubated with 10 μM BrdU (BromodeoxiUridne, an analog of thymidine) (B23151, Life Technologies) for 4–8 h. Cells were fixed in 70% (v/v) cold ethanol, permeabilized and incubated with anti-BrdU antibody (B35139, Life Technologies), which was diluted at 1:200 (v/v) in 1% (v/v) FBS. Cells were counterstained with DAPI as above. As a negative control, control cells were stained with anti-BrdU antibody without incubation in the BrdU solution. The absence of fluorescent signal indicated the specificity of the BrdU detection.

### Actin staining

To interrogate the actin organization of the spheroids of hepatoma compared to 2D cultures, the samples were fixed and permeabilized as above. The samples were incubated with 1:50 (v/v) phalloidin-Alexa594 (Life Technologies) and counterstained with DAPI (Sigma-Aldrich). The samples were imaged using confocal spinning microscopy (Nikkon, France) or the motorized fluorescent microscope.

### RT-PCR analysis

The total spheroids of a 2-day culture period were harvested from the chips, as described above. Alternatively, cells cultured on regular T-flasks were recovered using trypsin after the same cultivation time. The total RNA of 1 × 10^4^ cells were extracted and converted to cDNA using Superscript III CellsDirect cDNA synthesis System (18080200, Invitrogen, Life Technologies), following the manufacturer instructions. After cell lysis, a comparable quality of the extracted RNA was observed using a bleach agarose gel and similar RNA purity was obtained by measurement of the optical density at 260 and 280 nm using a NanoDrop spectrophotometer (Thermo scientific, Wilmington, DE, USA), between total RNA preparations from 2D and on-chip cultures.

The cDNA were amplified using GoTaqqPCR master mix (Promega, Charbonnieres, France) and primers for ALB and GAPDH (Life technologies, Saint Aubin, France) at the specified melting temperature (*T*_m_) (Supplementary Table 2), using a Mini-Opticon (Bio-Rad) thermocycler. As negative control, water and total RNA served as template for PCR. To validate the specificity of the PCR reaction, the amplicons were analyzed by dissociation curve and subsequent loading on a 2.5% (w/v) agarose gel and migration at 100 V for 40 min. The PCR products were revealed by ethidium bromide (Sigma-Aldrich) staining, and the gels were imaged using a trans-illuminator. The analysis of the samples non-subjected to RT^−^ indicated negligible genomic DNA contamination (i.e., <0.1%), while no amplification signal was observed for the water template (NTC). The amount of ALB transcripts was normalized to the endogenous reference (GADPH) (ΔCt), and the relative expression to a calibrator (2D cultures) was given by 2^−ΔΔCt^ calculation. A least three biological replicates of 2D and on-chip cultures were analyzed by at least duplicate measurements. The standard curves for GADPH and ALB were performed using a five serial dilution of the cDNA templates, and indicated almost 100% PCR efficiency (Supplementary Table 2).

### Dynamically regulated drug perfusion and live imaging

The microfluidic chip was equipped with two inlets at one end of the chamber. The chip was loaded with H4-II-EC3 cells one day before the perfusion, as previously described. The day of the experiment, the chip was incubated for 30 min with culture medium containing 2 drops per mL of NucBlue Live reagent (ReadyProbes, ThermoFischer) and 0.5 μM propidium iodide (Sigma-Aldrich), after oil to medium phase change.

Two different solutions were prepared for the perfusion culture. Each solution contained culture medium plus NucBlue Live reagent (1 drop per 10 mL), 0.5 μM propidium iodide, and 50 μL of antifoam C emulsion (Sigma-Aldrich); the solution containing the drug was additionally supplemented with 30 mM acetaminophen (Sigma-Aldrich) and 10 μM fluorescein (Sigma-Aldrich). 1 mL glass syringes (SGE, Analytical Science, France) were filled with each solution, while the two stock solutions were stored in 50 mL tubes. The two syringes were connected to bubble traps (Elvesys, Paris, France), and then plugged to the two chamber inlets.

The perfusion culture was controlled by neMESYS syringe pumps. At each time point, the drug solution was perfused at 10 or 30 μl min^−1^, while the other solution was injected at a flow rate enabling to maintain the sum of the two flow rates at 40 μl min^−1^. The flow rates were automatically changed every hour, which enabled three different perfusion conditions. Because the volume of medium needed to perform the experiment was much larger than the volume of the syringes, two additional 1 mL syringes were simultaneously refilled with the two stock solutions and at the same rate as for the injection. The syringes used for the perfusion were refilled every 30 min. At the same time, the refilled syringes with each of the two solutions were immediately used to maintain the continuous perfusion. This operation was automated using an electro-valve connected to each syringe pump unit, according to the manufacturer instructions. Moreover, air supplemented with 5% CO_2_ was continuously blown into the stock solutions, which were maintained in the microscope incubator at 37 °C. The spheroids are imaged every 30 min, as previously described.

### 2D/3D coculture on chip

The microfluidic chip was equipped with a ridge (50 μm width, 130 μm height), placed in the center of the culture chamber, which separated two distinct regions in the device. Six outlets bordered the ridge in parallel (three for each side of the ridge). After plasma treatment, a fibronectin solution (50 μg mL^−1^) was flushed into the first area of the chip, while this solution did not enter in the second part. The chip was left for 1 h to allow fibronectin coating on the ionized PDMS surface. Next, the second area of the culture chamber containing the anchors was treated with Novec, as described above. Importantly, the Novec never mixed with the fibronectin solution.

After the different coating treatments, the area coated with fibronectin was filled with culture medium. The droplets containing H4-II-EC3 were produced in the Novec-treated area, as described above. Finally, a 6 × 10^6^ BAECs mL^−1^ solution was filled into the fibronectin region. After overnight incubation, BAECs spread on the PDMS surface and spheroids were formed in the Novec-treated area.

Oil-to-medium phase change was performed in the Novec-treated area, as described above, and the co-culture was placed in a CO_2_ incubator for longer cultivation. Cell viability was assessed by a LIVE/DEAD staining kit (Molecular Probes, Life Technologies), after a 48 h culture period, as described above.

### 3D co-culture on chip

H4-II-EC3 and BAECs were harvested with trypsin at 60–70% confluence. BAECs were stained with CellTracker Red CMTPX (Life Technologies), according to the manufacturer instructions. Several mixes of the two populations containing a total of 1.2 × 10^6^ cells were prepared in 70 μL FBS-containing medium. The different mixes contained 0, 10, 20, 30, 50, 70, and 80% of BAECs for 100, 90, 80, 70, 50, 30, and 20% of H4-II-EC3, respectively. Then the solutions were mixed with a 30 μL 3% (w/v) liquid low melting agarose solution (i.e., stored at 37 °C) (Sigma-Aldrich, Saint Quentin Fallavier, France) diluted in culture medium (1:3 v/v), resulting in a 100 μL solution of 12 × 10^6^ cells mL^−1^ in 0.9% (w/v) agarose solution. About 50 aqueous droplets from each cell mixes were formed by flow focusing and captured in the capillary anchors, as above. After spheroid formation (about 24 h), the agarose was gelled at 4 °C for 30 min. After PEG-di-Krytox washing, all the cells forming the hetero-spheroids were stained with NucBlue Live reagent (Life Technologies).

### Image analysis

All the images were processed and analyzed using a custom Matlab code (R2014b, Mathworks, Natick, MA, USA). Two distinct routines were used: one for the spheroid formation process (Supplementary Fig. 5a) and one for the fluorescence experiments (Supplementary Fig. 6a).

For the monitoring of spheroid formation, the entire array was imaged every 20 min during 24 h in bright field (Supplementary Fig. 5b). The anchors were first detected in each image by convolution with a properly scaled hexagonal mask (Supplementary Fig. 5c), and tracked over time. Cells were detected in each of these anchors by first calculating the magnitude of the local intensity gradient at each pixel (Supplementary Fig. 5d), and then applying a watershed transformation. Sometimes, more than one object was detected within the anchors. Indeed, several aggregates can begin to form in one anchor, for instance one at the bottom of the droplet and one close to the anchor sidewalls. Most of the time, the latter ends up falling to the droplet bottom and merges with the other to form a single spheroid. In the pre-fusion images, the largest spheroid was selected as the spheroid of interest. Results of the image analysis were manually checked to detect possible inconsistencies in spheroid detection. Morphological data (area, diameter and shape index) were then computed for the spheroids over time. The shape index *ShI* was computed according to the following formula:

$${\rm ShI} = \frac{{4\pi A}}{{{P^2}}}$$

where *A* and *P* are, respectively, the area and perimeter of the cells as detected by the code. The diameter of a spheroid was calculated as the diameter of the disc with an equivalent area as the aggregate.

For all the fluorescence stainings (DAPI/ALB, DAPI/BrdU, or LIVE/DEAD), the entire array was imaged in bright field and with two fluorescent channels. As above, the anchor detection was performed by convolution on the bright field image. In each of the anchors, a fluorescence threshold was automatically chosen (Supplementary Fig. 6b) and the morphometric data on all the detected objects were stored. The fluorescence data at the object level were processed differently depending on the staining. For the LIVE/DEAD experiments the viability was defined as:

$$\begin{array}{*{20}{l}}{\rm{viability}}(\% ) = \frac{{{\rm{object}}\,{\rm{area}}\,{\rm{(pixels)}} - {\rm{number}}\,{\rm{of}}\,{\rm{pixels}}\,{\rm{above}}\,{\rm{the}}\,{\rm{TRITC(DEAD)}}\,{\rm{threshold}}}}{{{\rm{object}}\,{\rm{area}}\,{\rm{(pixels)}}}} \hfill \end{array}$$

For the DAPI and ALB stainings, a local background value was calculated in each anchor and for each fluorescence channel as the mean intensity of the pixels that did not correspond to an object (spheroids, cell aggregate, cell unit). The fluorescent signal of an object was obtained by subtracting this local background from the mean fluorescent intensity of its pixels. The morphometric data were then used to sort the objects into three different groups (Supplementary Fig. 6c): The first group was defined as “cell units”, which show a high variance and a diameter between 9 and 40 µm. The detected objects that had a diameter above 40 µm and a *ShI* below 0.5 were called “cell aggregates”. Finally, the “spheroids” were selected as the objects having a diameter above 40 µm and a *ShI* above 0.5. Objects that were too small (diameter<9 µm) or out of focus (low variance, calculated by combining the bright field image with the objects detection in fluorescence) were discarded from the analysis.

In the three types of fluorescence experiments, cellular level data were acquired by the detection of local fluorescence maxima. These maxima determined the cell location for the dead cells with the DEAD staining and for all the nuclei with the DAPI fluorescence, or the ALB peaks inside the spheroids.

The intensity of the ALB peaks was processed separately, to account for the volume effects (Supplementary Fig. 6d). Indeed, in spite of our low depth of field (about 5 μm with our objective, 10×, NA = 0.25) the fluorescent signal was polluted by out-of-focus fluorescence, resulting in a higher ALB signal in the central region of our spherical spheroids. To correct this, a local spheroid background was estimated as a function of *r*/*R* (Supplementary Fig. 6d, black line) by fitting the spheroid pixels that are not close to an ALB peak with a polynomial (*ax* ^3^+*bx* ^2^+*c*). This background, which was determined for each spheroid, was subtracted from the raw amplitude of the ALB peaks (Supplementary Fig. 6d, *blue dots*) to obtain the corrected ALB signal (Supplementary Fig. 6d, *green dots*). This corrected signal was then divided, for each spheroid, by the value of the spheroid background at *r*/*R* = 0 in order to minimize the inter-spheroid variability in a chip. Finally, this signal was centered on 1 for each chip by dividing each ALB peak corrected signal by the mean on the chip, in order to obtain comparable intra-spheroid variations from different chips (Fig. 3h and Supplementary Fig. 7c).

Finally, the BrdU signal was calculated in each nucleus (around the DAPI maxima) as well as a BrdU/DAPI signal ratio. The BrdU^+^ cell population was selected automatically in each experiment from the high values of the BrdU/DAPI signal ratio histogram (Supplementary Fig. 6e).

Image processing was also used to get quantitative data on 2D cultures (Fig. 2c, d), with LIVE/DEAD or DAPI/BrdU staining. The analysis was similar to the one performed for the microfluidic chip, without the anchor detection, the variance calculation nor the morphological group sorting. In the two cases, only one ratio value (viability or ratio of BrdU^+^/DAPI^+^ cells) was defined on all the detected cells of an image.

### Statistical analysis

35 chips were analyzed for a total amount of 10,113 spheroids. 11 chips were stained with LIVE/DEAD, 19 chips for DAPI and ALB and 5 chips for DAPI and BrdU. For the population-level comparison between 2D and 3D conditions (Fig. 2d–f), statistical significance was assessed by unpaired two-sample two-tailed Student’s *t*-tests. For the spheroid-level and cellular-level data analysis, significance was assessed either by Welch’s ANOVA followed by Games-Howell post hoc procedure (Fig. 3c, e, h) or a Welch’s *t*-test (Supplementary Fig. 9c, 11c). For non-normal distributions, significance was tested using Kruskal–Wallis ANOVA followed by Mann–Whitney *U*-tests with Sidak’s correction for multiple comparisons (Fig. 3a, b, Fig. 5c, e and Supplementary Fig. 10). *p < 0.05, **p < 0.01, ***p < 0.001, were considered statistically significant. N.S.: non-significant. *p*-value ranges are only indicated for the highlighted comparisons.

In the Tukey box-and-whiskers figures (Fig. 3b, c, e, h and Fig. 5c, e), the boxes represent the first (q1) and third (q3) quartiles with the median shown by the line bisecting the box, and the mean is shown with black circles. The whiskers represent 1.5 times the inter-quartile range (q3–q1) of the sample. Finally, the box width is proportional to $\sqrt n$ .

### Data availability

Data supporting the findings of this study are available within the article (and its Supplementary Information files) and from the corresponding author upon reasonable request.

## Electronic supplementary material

Supplementary Information

Supplementary Movie 1

Supplementary Movie 2

Supplementary Movie 3

Supplementary Movie 4

Supplementary Movie 5
