# Supplementary material for: Multiscale cytometry and regulation of 3D cell cultures on a chip
Source: Nat Commun. 2017 Sep 7;8:469. doi: 10.1038/s41467-017-00475-x (PMC5589863; doi:10.1038/s41467-017-00475-x)
Supplement: Supplementary file 1 — Supplementary Information [file 41467_2017_475_MOESM1_ESM.pdf]

Description of Supplementary Files

File Name: Supplementary Information
Description: Supplementary Figures and Supplementary Tables

File Name: Supplementary Movie 1
Description: Follow-up of a droplet from the production to the trapping. Approximately 200 H4-II-EC3 cells, suspended in 1 % (w/w) agarose, are encapsulated in 16 nL droplets. The oil flow drives the droplets through the rails to the trapping chamber where they are immobilized. Time is shown in min:sec.

File Name: Supplementary Movie 2
Description: Chip loading. The droplets enter the chamber and are evenly distributed across the chamber width thanks to capillary rails. Time is shown in min:sec.

File Name: Supplementary Movie 3
Description: Image analysis of the spheroid formation in one anchor. Images are taken every 20 min for 24 hours while keeping the chip at 37 °C. The red border shows the detected edges of the sedimented cellular aggregate. The left panel shows the real time evolution of the normalized area and shape index of the detected object.

File Name: Supplementary Movie 4
Description: Oil to medium phase change around the spheroids. After 24 hours at 37 °C, each droplet contains a single spheroid in a liquid agarose and are surrounded by oil. After gelation at 4 °C, the culture medium is introduced in the chamber and replaces the oil, merging with the solidified agarose beads. Time is shown in min:sec.

File Name: Supplementary Movie 5
Description: Selective spheroid extraction. After the oil to medium phase change, a single spheroid is extracted from the array by melting the agarose capsule using localized infrared laser (visible as a bright spot on the movie). After the agarose melting, the spheroid is carried out of the chip by the medium flow. Time is shown in min:sec.

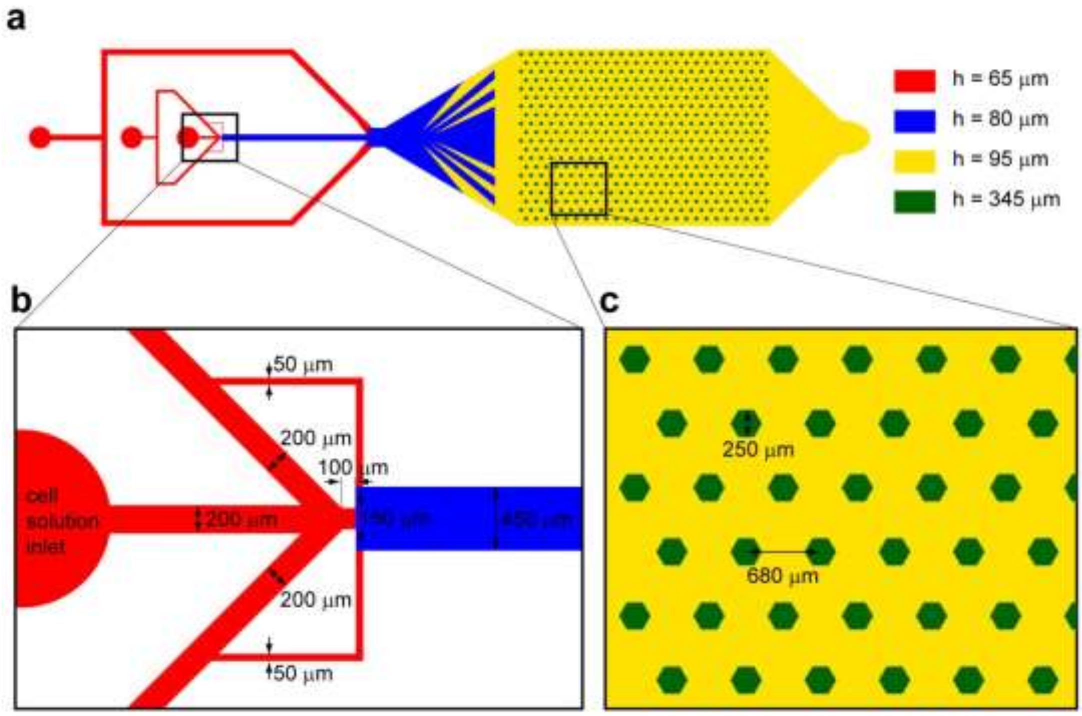

Supplementary Figure 1 | Schematic view with dimensions of the whole chip (**a**), the junction (**b**) and the anchors (**c**).

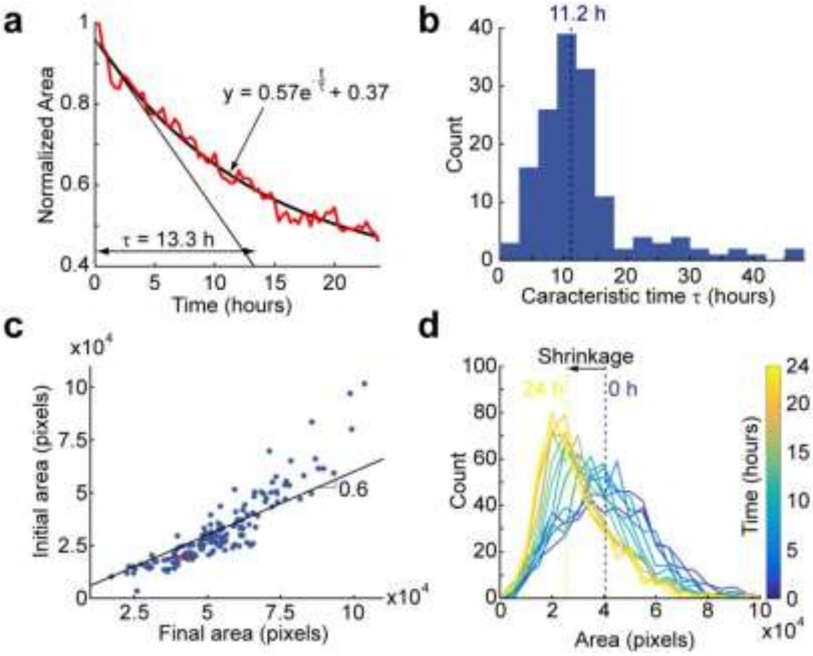

Supplementary Figure 2 | Analysis of the spheroid formation. (**a**) Evolution of the normalized area of the spheroid displayed in **Fig.1.g** over time. The black curve shows an exponential fit, with a characteristic time τ of 13.3 hours. (**b**) Histogram of the characteristic shrinking time for the analyzed spheroids n = 152 spheroids). The dashed line shows the median of the data. (**c**) Initial projected areas versus the final areas after the spheroid formation. Each dot corresponds to one spheroid, while the red dot is the spheroid showed in **Fig.1.g**. Most of the spheroids had a shrinking rate close to 0.6. (**d**) Area histogram of the 500 detected spheroids over time. The blue and yellow dashed lines show respectively the median area at t = 0 h and t = 24 h.

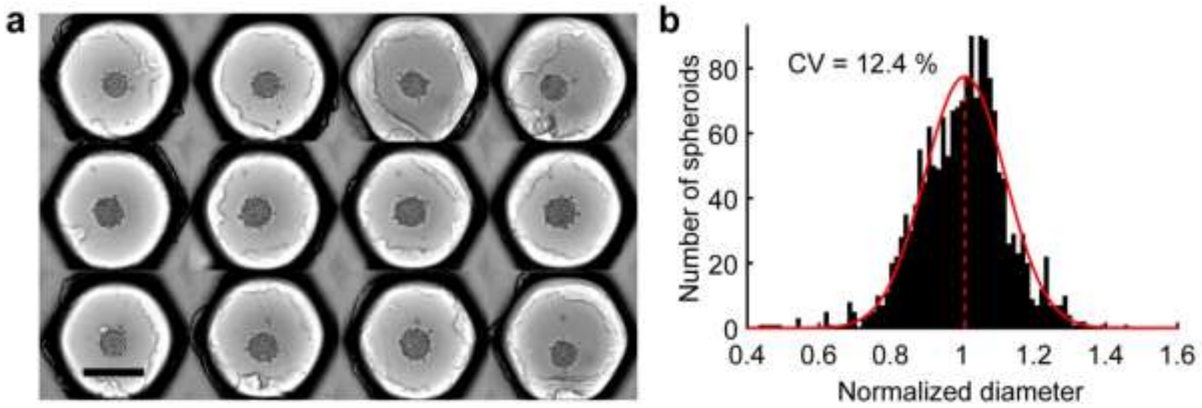

Supplementary Figure 3 | Spheroids in 400 µm anchors. (**a**) Montage showing 12 spheroids formed in anchors with a 400 µm diameter (252 anchors in a single chamber). The volume of the trapped droplet is about 60 nL and the cell concentration is 6.10^6 cells.mL^{-1}. The overall mean diameter is 104.5 µm. Scale bar is 200 µm. (**b**) Distribution of the spheroid diameter normalized for each chip (7 chips, n = 1,701 spheroids). The red line represents a Gaussian fit of the data.

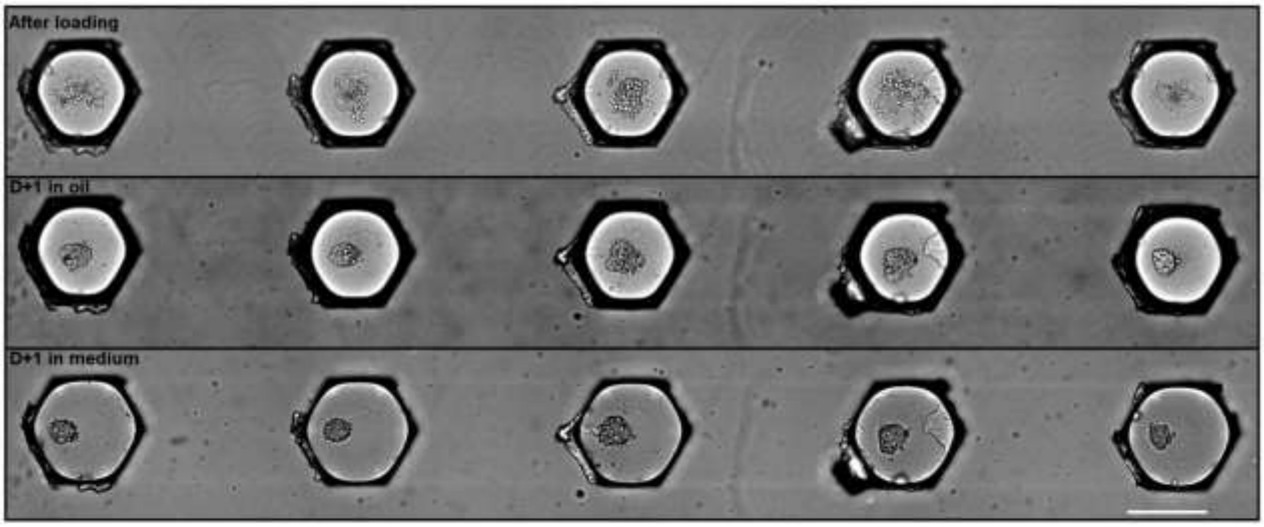

Supplementary Figure 4 | Time lapse images of 5 anchors during the change of external phase from oil to aqueous. After loading, the cells sedimented at the bottom of each droplet. One day later (D+1 in oil), one spheroid was formed in each anchor. These spheroids stayed in place (D+1 in medium) during the phase change. Scale bar is 200 µm.

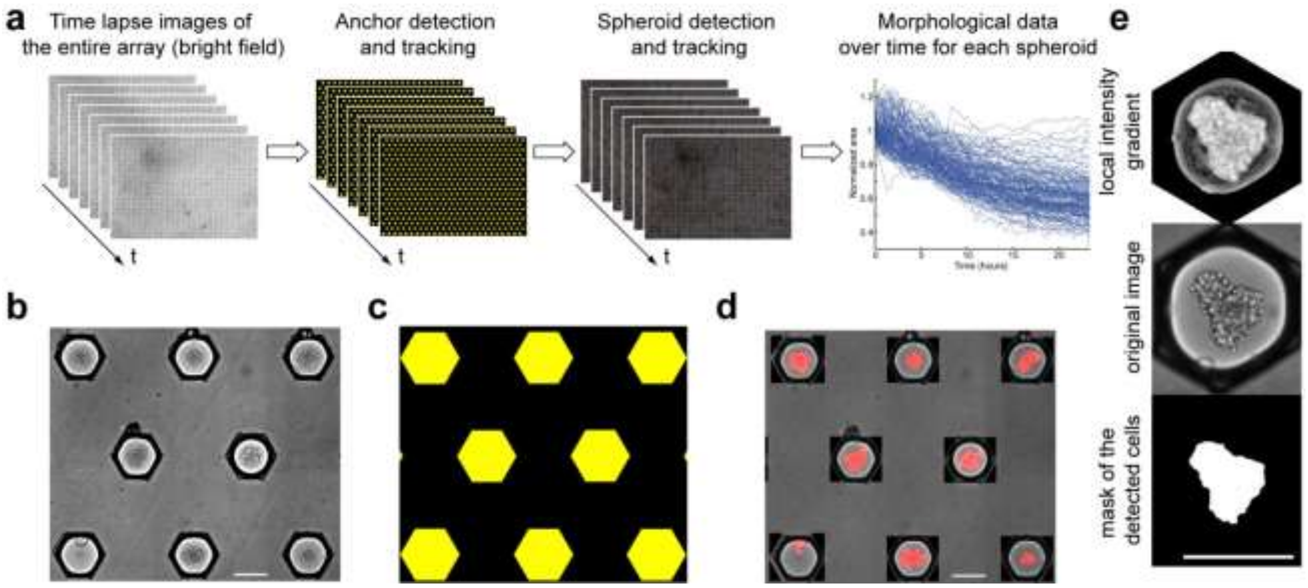

Supplementary Figure 5 | Image analysis of the spheroid formation (bright field). (**a**) Method for the image analysis of the spheroid formation. A picture of the entire array is taken in bright field every 20 minutes for 24 hours (**b**). The anchors are detected in all the images and tracked over time (**c**). In each anchor, the spheroid boundaries are detected (**d**, highlighted in red) based on the local intensity gradient (**e**, top). Scale bar are 200 µm. See online methods for detailed description.

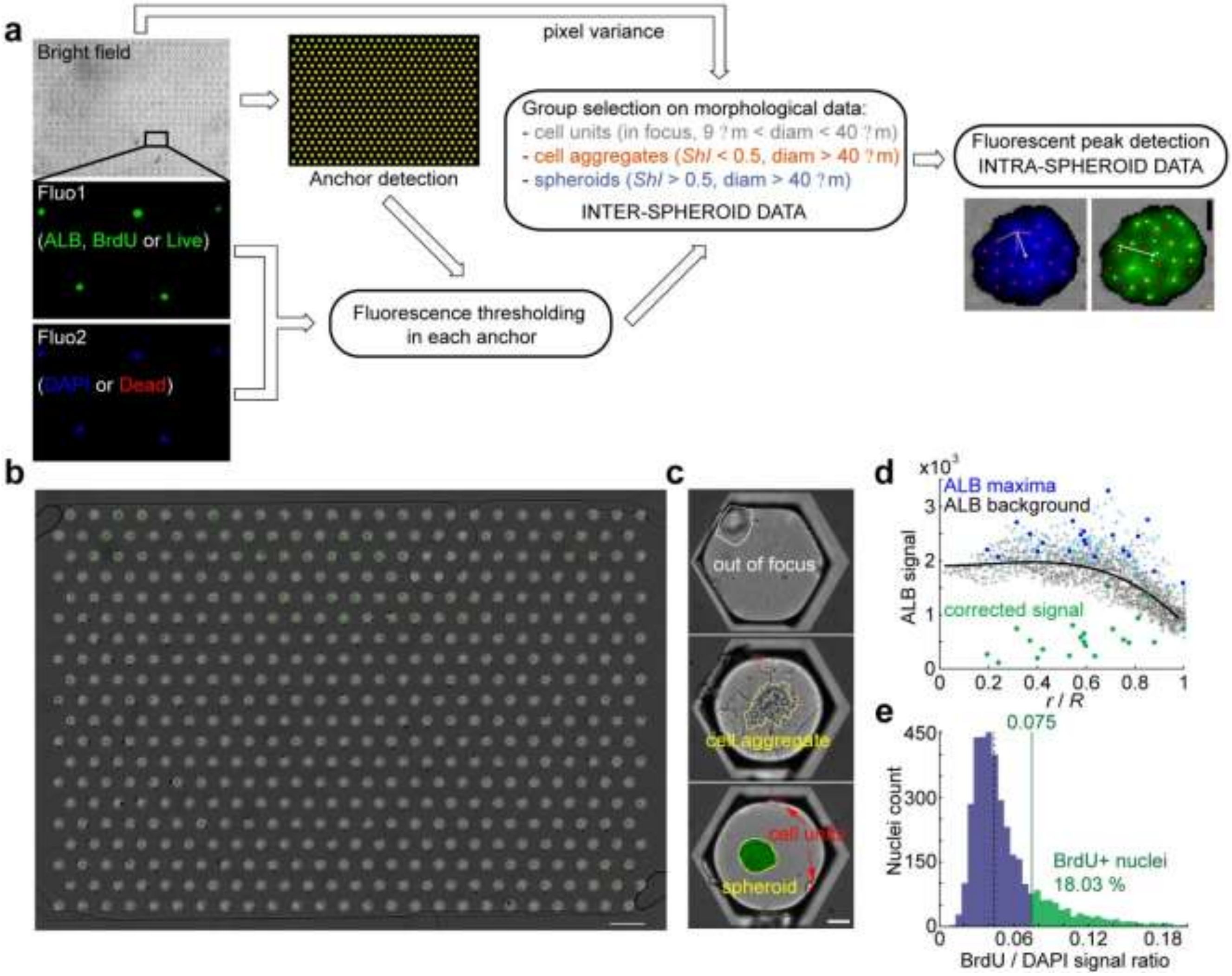

Supplementary Figure 6 | Fluorescence image analysis. (**a**) Method for the image analysis of a fluorescence experiment. An image of the entire array is taken in bright field and with two fluorescent channels. The anchors are detected on the bright field image, allowing an automatic thresholding for the fluorescent channels in each anchor (**b**, scale bar is 1 mm). This detection, with variance calculations, allows for sorting the objects among the different groups based on morphological criteria (**c**, scale bar is 50 µm). The intra-spheroid data are acquired by fluorescence peak detection in each spheroid. (**d**) Normalization of the ALB signal in one spheroid (diameter 68.8 µm, 1,450 pixels, 16 detected nuclei and 21 detected ALB peaks). The ALB signal is represented for the ALB peaks (●) and for all the other spheroid pixels (+ : the closest peak neighbors, + : other pixels) regarding *r / R* . The local spheroid background is obtained by a polynomial fit (―) of the pixels not too close to an ALB peak (+) and is subtracted from the raw signal of the ALB peaks to obtain the corrected ALB signal (●). (**e**) BrdU / DAPI signal ratio histogram for one experiment (n = 3,832 nuclei in 258 spheroids). The BrdU^{+} cell population is highlighted in green. --- : median of the distribution. See online methods for detailed description.

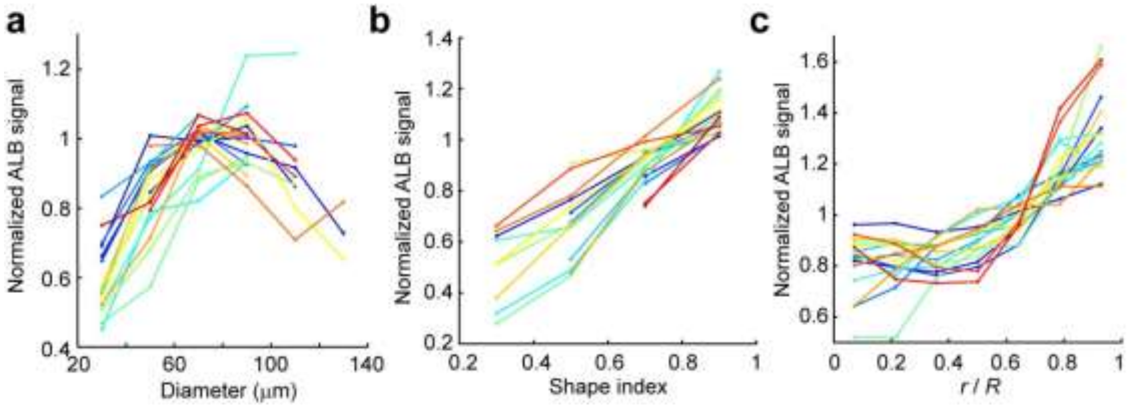

Supplementary Figure 7 | Trends per chip for ALB. (**a-b**) Evolution of the mean of the normalized ALB signal with the spheroid diameter (**a**) and the shape index (**b**). (**c**) Evolution of the normalized ALB signal at the detected maxima with the normalized distance to the spheroid center. Each curve represents one microfluidic chip.

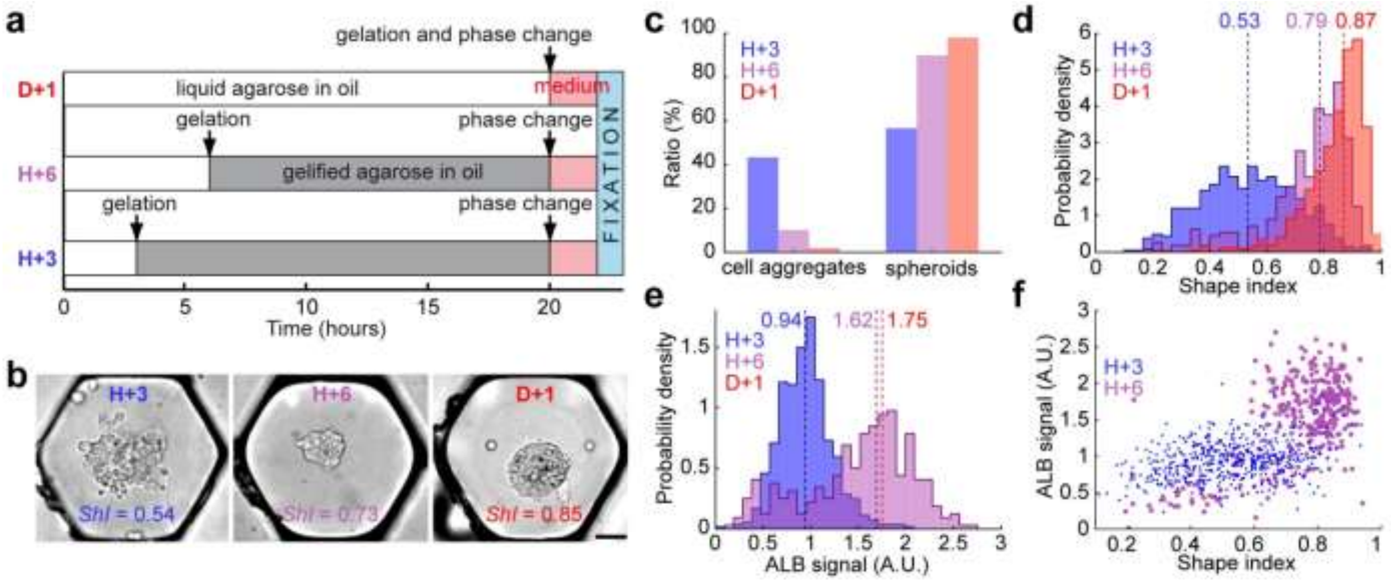

Supplementary Figure 8 | Blockage of the spheroid formation. (**a**) 2 experiments were carried out with different gelation times to show the effect of a disrupted spheroid formation. In the control experiments (D+1, red, n_1 = 1,712 spheroids, n_2 = 34 cell aggregates, 5 chips) the cells were allowed to reorganize overnight in liquid agarose. The agarose was gelled 20 hours after the droplet loading and the external oil was replaced by culture medium. In the blocking experiments, the spheroid formation was disrupted by gelling the agarose 6 hours (H+6, purple, n_1 = 301 spheroids, n_2 = 34 cell aggregates, 1 chip) or 3 hours (H+3, blue, n_1 = 311 spheroids, n_2 = 238 cell aggregates, 1 chip) after the cell loading. The external phase change always occurred 20 hours after the beginning of the experiment. The cells are then fixed prior to fluorescent staining and image analysis. LIVE/DEAD® staining on the blockage experiments showed no observable effect on cell viability. (**b**) Bright field images of one spheroid per condition, with a representative shape index value. Scale bar is 50 µm. (**c**) Evolution of cell aggregates to spheroids ratio. Evolution of the shape index (**d**) and of the ALB signal (**e**) with the gelation time. The dashed lines (**d-e**) show the median of the corresponding data. The red dashed line in e shows the mean ALB signal value for the D+1 experiments (see **Fig.3.e**). (**f**) The ALB signal increased with the shape index in the two blockage experiments.

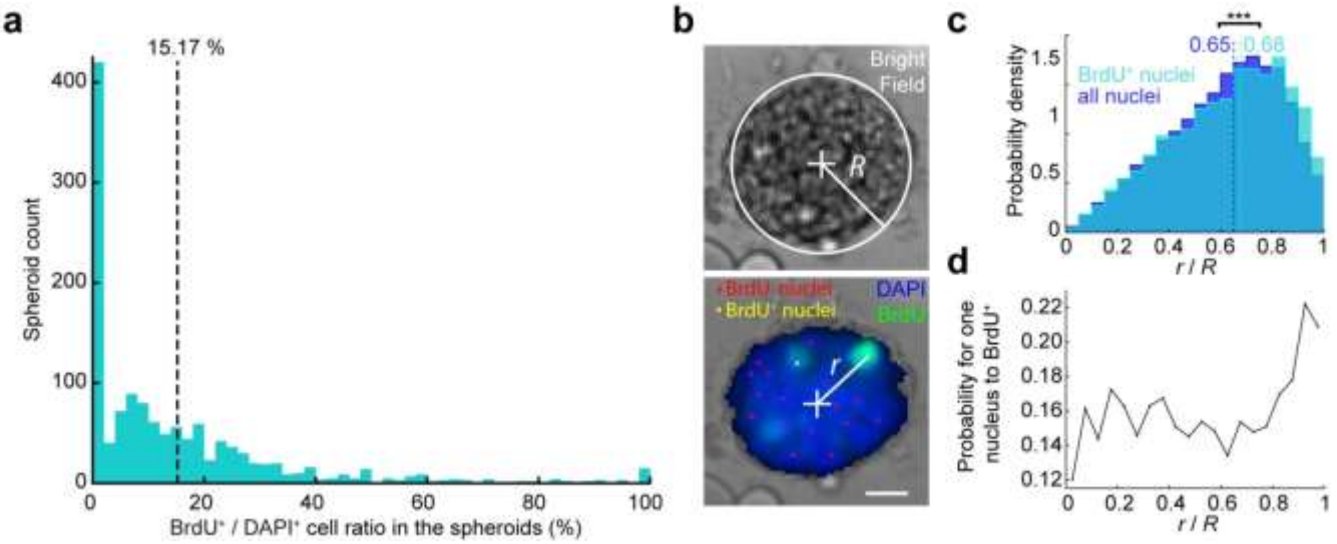

Supplementary Figure 9 | Quantitative analysis of the spheroid proliferation. (**a**) Histogram of the BrdU$^+$ / DAPI$^+$ cell ratio in the spheroids. – – : mean BrdU$^+$ / DAPI$^+$ ratio. (n = 1,311 spheriods, 5 chips). (**b**) Image analysis on a spheroid of radius *R*, shown in bright field (top) and stained with DAPI (blue) and BrdU (green) (bottom). Each detected nucleus center (DAPI$^+$) is highlighted with a red pixel, while those which are BrdU$^+$ nuclei (cyan, n = 4,154 BrdU$^+$ nuclei) and all the detected nuclei (n = 25,595 nuclei). The dashed lines show the median of each distribution. (**d**) The probability for one nucleus to be BrdU$^+$ can be calculated at each *r / R* value from the data in **c** and increases at the edge of the spheroid.

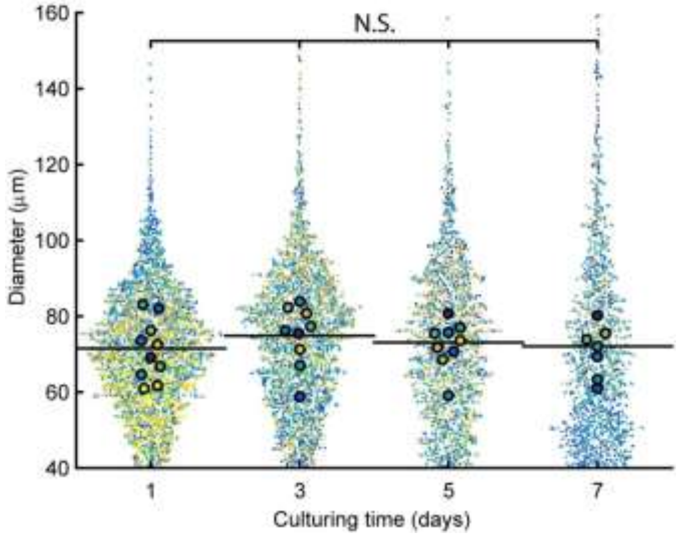

Supplementary Figure 10 | Violin plot of the spheroid diameter evolution over time. For each time point, one color corresponds to one chip. Large colored dots show the mean while the smaller colored dots represent individual spheroids. Each time point mean is represented by a black line. D+1: n = 10 chips; D+3: n = 9 chips; D+5: n = 9 chips; D+7: n = 7 chips; overall number of spheroids = 10,113.

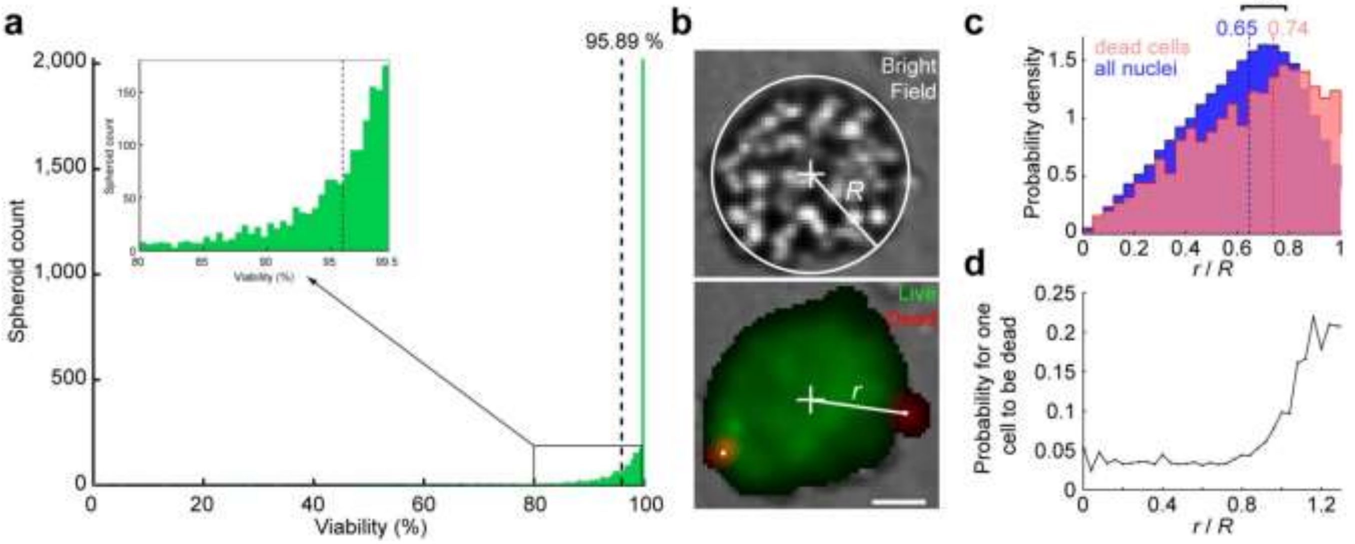

Supplementary Figure 11 | Quantitative analysis of the spheroids viability. (**a**) Histogram of the spheroid viability (see viability formula in online methods). – – : mean spheroid viability. (n = 3,877 spheroids, 11 chips). The insert shows the part of the histogram between 80 % and 99.5 % viability. (**b**) Image analysis on a spheroid of radius *R*, shown in bright field (top) and stained with LIVE/DEAD® (bottom). Each dead cell center is detected (white pixel) and its distance *r* to the spheroid center is calculated. Scale bar is 20 µm. (**c**) Location probability density for the dead cells (red, n = 3,634 dead cells) and all the detected nuclei with the DAPI experiments (n = 125,734 nuclei). The dashed lines indicate the median of each distribution. (**d**) The probability for one cell to be dead can be calculated at each *r* / *R* value from the data in **c** and increases at the edge and at the exterior (*r* / *R* > 1) of the spheroid.

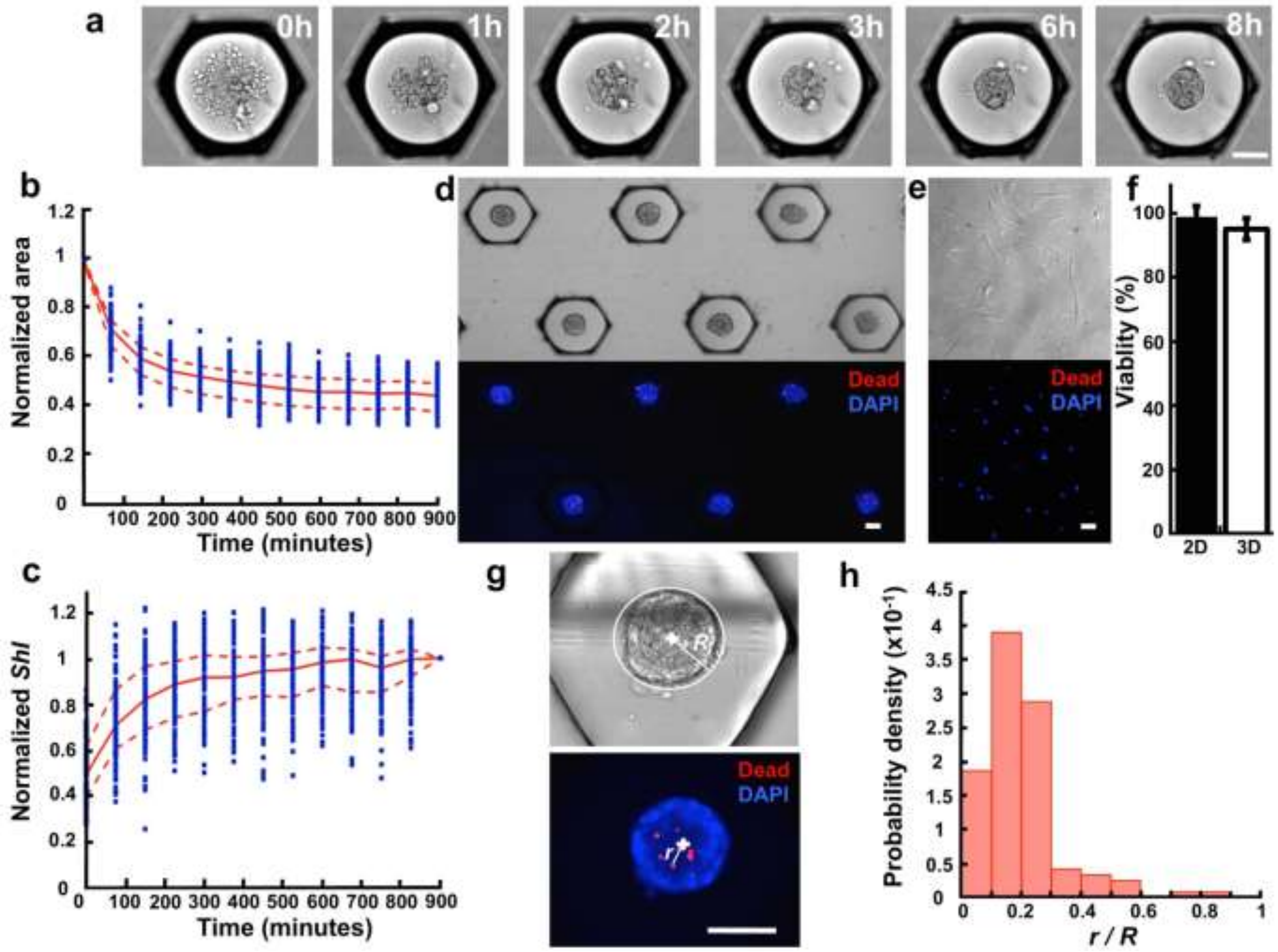

Supplementary Figure 12 | Formation of spheroids from primary human mesenchymal stem cells. Time lapse of spheroid formation in one anchor (**a**). Dynamics of the spheroid formation: normalized area (**b**) and normalized shape index (*ShI*) (**c**) evolution (n = 100 spheroids). Each blue dot represents one spheroid, red line = median, dashed red lines : first and third quartiles. Representative images of spheroids in the anchors (**d**) and 2D cells (**e**) stained for live/dead (ReadyProbes®) (**c**). Comparison of the viability of the spheroids on chip and of the cells in 2D (**f**). Image analysis on a spheroid of radius R, shown in bright field (top) and stained with live/dead (bottom)(**g**). Each dead cell center is detected and its distance r to the spheroid center is calculated. (**c**) Location probability density for the dead cells and all the detected nuclei with the DAPI experiments. Scale bar is 100 µm.

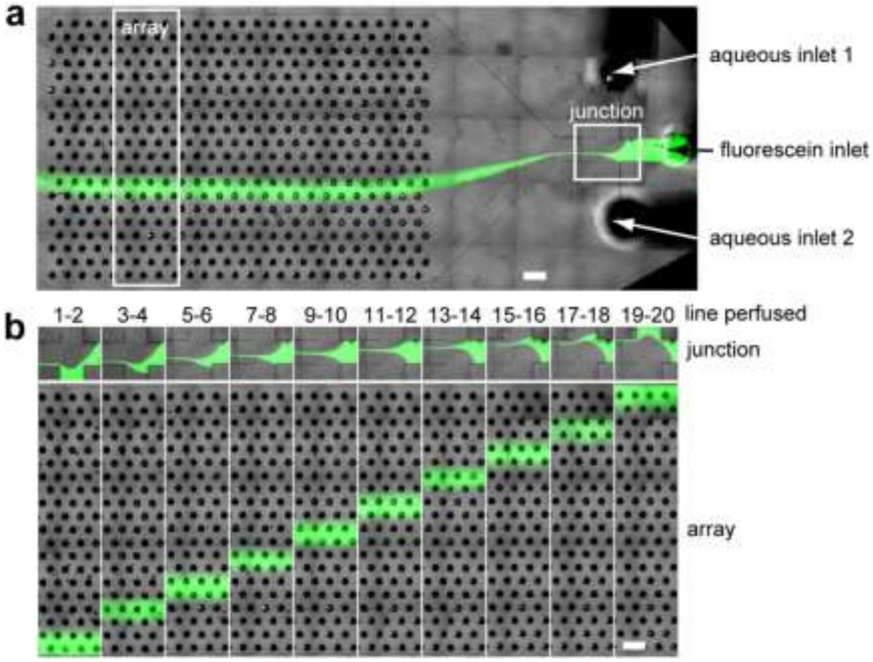

Supplementary Figure 13 | Line by line perfusion of the array. (**a**) Micrograph of the entire anchor array showing the three inlets added at the end of the chamber. A fluorescein solution and an aqueous buffer flow respectively through the middle and side inlets. The white rectangles represent the location of the images shown in (**b**). (**b**) The first row of images represents the junction of the three aqueous inlets while the second row shows a portion of the array. Each column stands for different flowrate leading to different lines perfused with the fluorescein solution. Scale bars are 1 mm.

Supplementary Table 1 | Flow rate for cell loading and phase exchange.

**Step**	**Liquid loader**	**Flow rate (µL.min^{-1})**	**Approximate delivered volume (µL)**
Cell loading	Chamber	35	105
	Junction	5.8-6.2	20
	Cells	5	15
Surfactant washing	Exit of the chamber	40	2000
Culture medium	Exit of the chamber	4	50

Supplementary Table 2 | Primer sequences.

**Gene**	**Orientation**	**Sequence**	**Product size (bp)**	**Tm (°C)**	**qPCR efficiency**
Albumin	Forward	GTGTTTCCTGCAGCACAAGG	789	57	103 %
	Reverse	TCAGCACAGCACTTCTCCAG			
GAPDH	Forward	TGTGAACGGATTTGGCCGTA	208	60	98.6 %
	Reverse	GATGGTGATGGGTTTCCCGT			
